# Group A, B, C, and G *Streptococcus* Lancefield antigen biosynthesis is initiated by a conserved α-d-GlcNAc-β-1,4-l-rhamnosyltransferase

**DOI:** 10.1074/jbc.RA119.009894

**Published:** 2019-09-10

**Authors:** Azul Zorzoli, Benjamin H. Meyer, Elaine Adair, Vladimir I. Torgov, Vladimir V. Veselovsky, Leonid L. Danilov, Dusan Uhrin, Helge C. Dorfmueller

**Affiliations:** ‡ Division of Molecular Microbiology, School of Life Sciences, University of Dundee, Dundee, DD1 5EH, United Kingdom; § EaStCHEM School of Chemistry, University of Edinburgh, Edinburgh, EH9 3FJ, United Kingdom; ¶ N. D. Zelinsky Institute of Organic Chemistry, Russian Academy of Sciences, Moscow 119334, Russia

**Keywords:** Streptococcus pyogenes, glycosyltransferase, carbohydrate biosynthesis, bacterial genetics, nuclear magnetic resonance (NMR), surface polysaccharide, carbohydrate biosynthesis, glycosyltransferase, group A carbohydrate (GAC), Lancefield, rhamnosyltransferase, virulence factor

## Abstract

Group A carbohydrate (GAC) is a bacterial peptidoglycan-anchored surface rhamnose polysaccharide (RhaPS) that is essential for growth of *Streptococcus pyogenes* and contributes to its ability to infect the human host. In this study, using molecular and synthetic biology approaches, biochemistry, radiolabeling techniques, and NMR and MS analyses, we examined the role of GacB, encoded in the *S. pyogenes* GAC gene cluster, in the GAC biosynthesis pathway. We demonstrate that GacB is the first characterized α-d-GlcNAc-β-1,4-l-rhamnosyltransferase that synthesizes the committed step in the biosynthesis of the GAC virulence determinant. Importantly, the substitution of *S. pyogenes gacB* with the homologous gene from *Streptococcus agalactiae* (Group B *Streptococcus*), *Streptococcus equi* subsp. *zooepidemicus* (Group C *Streptococcus*), *Streptococcus dysgalactiae* subsp. *equisimilis* (Group G *Streptococcus*), or *Streptococcus mutans* complemented the GAC biosynthesis pathway. These results, combined with those from extensive *in silico* studies, reveal a common phylogenetic origin of the genes required for this priming step in >40 pathogenic species of the *Streptococcus* genus, including members from the Lancefield Groups B, C, D, E, G, and H. Importantly, this priming step appears to be unique to streptococcal ABC transporter–dependent RhaPS biosynthesis, whereas the Wzx/Wzy-dependent streptococcal capsular polysaccharide pathways instead require an α-d-Glc-β-1,4-l-rhamnosyltransferase. The insights into the RhaPS priming step obtained here open the door to targeting the early steps of the group carbohydrate biosynthesis pathways in species of the *Streptococcus* genus of high clinical and veterinary importance.

## Introduction

*Streptococcus pyogenes* is a versatile Gram-positive bacterium that infects only humans and is responsible for a remarkable number of mild to severe illnesses. At least 700 million individuals are affected each year worldwide by diseases as varied as impetigo, pharyngitis, scarlet fever, necrotizing fasciitis, meningitis, and toxic shock syndrome, among others (1–4). Moreover, autoimmune postinfection sequelae with high mortality rates, such as acute rheumatic fever, acute glomerulonephritis, or rheumatic heart disease, can affect individuals who had previously suffered from Group A *Streptococcus* infections, extending the list of clinical manifestations caused by this severe pathogen (5, 6).

As suggested by its diverse clinical manifestations, *S. pyogenes* rely on different mechanisms to withstand the host's defenses (7–11). These mechanisms are supported by the synthesis of a wide array of virulence factors, among which is the Group A carbohydrate (GAC),^4^ a surface polysaccharide that constitutes between 40 and 60% of the bacterial cell wall (12–15). GAC is composed of a [→3)α-Rha(1→2)α-Rha(1→] rhamnose polysaccharide (RhaPS) backbone with a β-d-GlcNAc (1→3) side chain modification on every α-1,2–linked rhamnose (Rha) (15–17). Recent structural examinations and composition analysis of the GAC also suggest the presence of glycerol phosphate (GroP) (18), an observation that remained unnoticed for over 50 years (19, 20). Further, Edgar *et al.* (18) demonstrated that ∼25% of GAC side-chain GlcNAcs are decorated with GroP, imparting a negative charge to this polymer that has implications for *S. pyogenes* biology and defense mechanisms (19, 21). This feature, previously identified in other surface glycans (22, 23), provided new insight into the structural composition, biosynthesis, and function of GAC.

GAC is proposed to be synthesized by 12 proteins, GacABCDEFGHIJKL, encoded in one gene cluster (*i.e.* MGAS5005_*spy0602–0613*) that has been found in all *S. pyogenes* species identified so far (7, 24). Through sequencing of transposon mutant libraries, Le Breton *et al.* (10, 25) discovered that eight of these genes, *gacABCDEFG* and *gacL*, are essential for *S. pyogenes* survival. This information supports the observation by van Sorge *et al.* (7), who identified via insertional mutagenesis that the first three genes of the cluster (*gacABC*) are essential.

**SCHEME 1.**
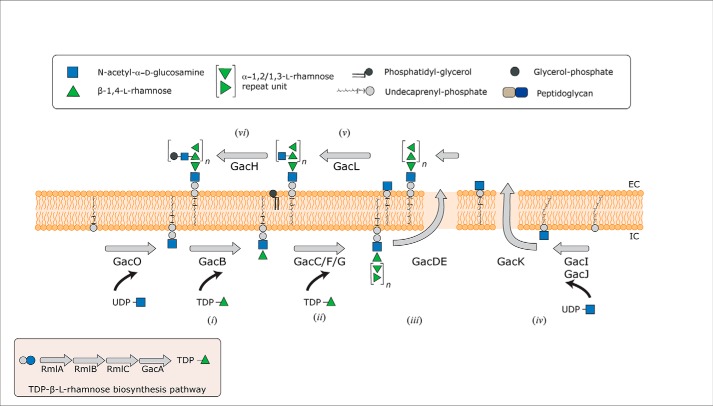
**Current model for the GAC biosynthesis pathway from *S. pyogenes*, Group A *Streptococcus*.**

The present hypothesis is that the GAC is formed in six consecutive steps, depicted in Scheme 1: (i) lipid-linked acceptor initiation, (ii) [→3)α-Rha(1→2)α-Rha(1→] RhaPS backbone synthesis, (iii) membrane translocation, (iv) synthesis of sidechain precursor, (v and vi) post-translocational chain modification steps. This product is linked to peptidoglycan (15). The cytoplasmic pool of TDP-β-l-rhamnose (TDP-Rha) necessary for RhaPS backbone synthesis is supplied by enzymes encoded in two separate gene clusters *rmlABC* and *gacA*/*rmlD* (24). Step (i) involves the addition of a Rha residue to a GlcNAc-PP-undecaprenyl (Und, WT_2_C_8_-). Rush *et al.* proposed that either GacB or GacC are a rhamnosyltransferase that transfers the first Rha residue onto the 3-OH group of the GlcNAc-PP-Und (14). The formation of the [→3)α-Rha(1→2) α-Rha(1→] RhaPS backbone constitutes the elongation step (ii), presumably achieved at the inner leaflet of the membrane by the glycosyltransferases (GTs) GacC, GacF, and GacG (14). However, the precise role of each GTs in the synthesis of the RhaPS backbone remains unknown. Consistent with this hypothesis are findings related to the biosynthesis of the *Streptococcus mutans* serotype C carbohydrate (SCC, previously referred to as RGP). Shibata *et al.* (26) reported that the genes *rgpA*/*sccB*, *rgpB*/*sccC*, and *rgpF*/*sccG* of this dental pathogen (homologs of *gacB*, *gacC*, and *gacG,* respectively) are required for the biosynthesis of the RhaPS backbone, although the structural or mechanistic examination of these enzymes was never conducted. According to the current gene annotation, the translocation step (iii) is proposed to be catalyzed by an ATP-dependent ABC transporter encoded by *gacD* and *gacE* (7, 14, 15). Concerning the post-translocational RhaPS modifications (v, vi), recent insights revealed that GacI synthesizes the sugar donor precursor GlcNAc-P-Und (iv) and that GacJ forms a complex with GacI to enhance the catalytic efficiency of this process (14). The lipid-linked monophosphate sugar GlcNAc-P-Und is proposed to be flipped across the membrane by GacK (14, 15). Furthermore, GacL was shown to utilize the extracellular GlcNAc-P-Und by transferring the GlcNAc onto the RhaPS backbone, to insert the antigenic β-d-GlcNAc (1→3) side chain modification on every α-1,2-linked rhamnose. Finally, GacH is a GroP-transferase that decorates the RhaPS-GlcNAc side chain (18). Enzymes conducting the transfer of GAC to the peptidoglycan as well as its linkage, presumably through phosphodiester bond (18), remain to be elucidated.

Often, the production of bacterial cell wall structures determines the fate of a bacterium in a given environment. Glycopolymers in particular, such as cell wall teichoic acids and capsular polysaccharides, are involved in growth, cell division, and bacterial homeostasis (15, 27–34). In the context of human health, these glycopolymers have been shown to play a significant role in biofilm formation, antibiotic resistance, host cell adhesion, and resistance to the host immune response by, for example, blocking antibacterial peptides and evading phagocytosis (8, 24, 35–39).

Many species within the *Lactobacillales* order present a surface RhaPS intertwined with additional components of the thick cell wall that is typical of Gram-positive bacteria. These surface glycans, as with other critical peptidoglycan-anchored structures, are essential for cell viability and play an essential role in pathogenesis (7, 9, 15, 24, 36, 40–42). In the *Streptococcus* genus, in particular in *S. pyogenes*, *S. agalactiae*, *and S. mutans*, genetic studies conducted on *S. pyogenes* strains showed that the absence of rhamnose glycopolymers results in severe growth defects associated with an altered cell wall and an increased susceptibility to muralytic enzymes (7, 25, 43, 43–45). Moreover, *in vivo* studies revealed that RhaPS depletion or its modification reduced the ability of *S. pyogenes*, *S. agalactiae*, and *S. mutans* to evade the immune system and attenuated their virulence (7, 25, 46–49).

Based on its essentiality, its role in pathogenesis, and the current evidence of the importance of RhaPS in bacterial homeostasis, we are confident that the detailed elucidation of all steps in the GAC biosynthesis pathway will play a decisive role in the development of antimicrobial drugs and anti-streptococcal vaccines in the near future (7, 46, 50). Despite the recent progress, several pressing questions remain unanswered regarding the biosynthesis of GAC. For example, the products of six of the 12 genes that constitute the GAC cluster (*gacBCDEFG*) have not yet been characterized, including the essential steps of GAC initiation, RhaPS backbone biosynthesis, and GAC translocation.

As a means of attaining more information on the GAC initiation step, we conducted an in-depth examination of the second enzyme encoded in the GAC gene cluster. Here we demonstrate that GacB, in disagreement with its preliminary genetic annotation and currently proposed mechanism of action (14), is the first rhamnosyltransferase in this pathway that transfers l-rhamnose from TDP-β-l-rhamnose onto the acceptor GlcNAc-PP-Und. GacB thereby forms a β-1,4-glycosidic bond between rhamnose and the lipid-linked GlcNAc through a metal-independent mechanism. Strikingly, our research on phylogenetically related homologs from other important human pathogenic streptococci, in particular from the Lancefield Groups B, C, and G, reveals that the role of GacB is conserved within the *Streptococcus* genus, suggesting a common first committed step for the production of RhaPS from all Lancefield groups. Thus, the implication of this research is relevant for those seeking to understand the pathogenesis and potential therapeutic interventions against streptococcal species important to human and animal health.

## Results and discussion

### Protein sequence analysis of the putative rhamnosyltransferase GacB

The current gene annotation indicates that the second gene of the GAC cluster encodes a putative α-d-GlcNAc-α-1,2-l-rhamnosyltransferase, named GacB (7, 15). Recent findings revealed that GlcNAc-PP-Und is essential for the GAC RhaPS backbone biosynthesis in *S. pyogenes* (14), supporting the current hypothesis that the addition of rhamnose during the initiation step occurs directly onto this lipid-disphosphate sugar acceptor (7, 15, 18). The *gacB* gene product is a 384-amino-acid-long polypeptide with a theoretical molecular mass of 44 kDa; this is consistent with the size of other known single-reaction GTs (51, 52).

*In silico* topological predictions conducted with SpOctopus- and TMHMM-based algorithms (53–55) indicate that GacB does not contain a signal peptide sequence but possesses a short hydrophobic region at its N terminus end, suggesting that this protein is not secreted but is tethered to the cytosolic side of the membrane (Fig. S1). This prediction is in agreement with the proposed function catalyzing the reaction onto a lipid-linked acceptor, as well as with the observed membrane association during the heterologous protein expression.

The most relevant structural information provided by the domain architecture analysis is that GacB belongs to the RfaB superfamily of GTs, a group of nucleotide sugar-dependent enzymes known to be involved in cell wall biosynthesis (54, 55). In the absence of any known function, GacB has been preliminarily classified based on its protein sequence into the GT4 family, a group of GTs with diverse biological functions with a characteristic GT-B type fold. The enzymes composing this group act through a retaining catalytic mechanism, meaning that the resulting stereochemistry at the anomeric carbon is conserved between the sugar donor and the product (56–61).

The sequence analysis revealed that GacB has two conserved domains, the GT_1_like6 domain covering the entire primary sequence (residues 1–384) and the domain of unknown function DUF1972 at the N terminus (residues 1–185). The DUF1972 domain is widely spread among glycosyltransferases (and rhamnosyltransferases) but has no defined functionality (55).

GacB shares 68% sequence amino acid identity and the same type of predicted domains as *S. mutans* SccB (Table 1), which has been proposed to initiate the biosynthesis of the SCC in *S. mutans*, a polysaccharide that also contains a RhaPS backbone (14, 26, 47, 62). Importantly, SccB also requires GlcNAc-PP-Und as an acceptor, as the biosynthesis of the surface polysaccharide is abrogated in an *E. coli* Δ*wec*A deletion strain (26, 56). WecA is the enzyme responsible for the GlcNAc-PP-Und biosynthesis in *E. coli*. GacO is the functional homolog of WecA in *S. pyogenes*, whereas *S. mutans* and several other streptococci contain similar homologs (26, 32, 63).

**Table 1:**
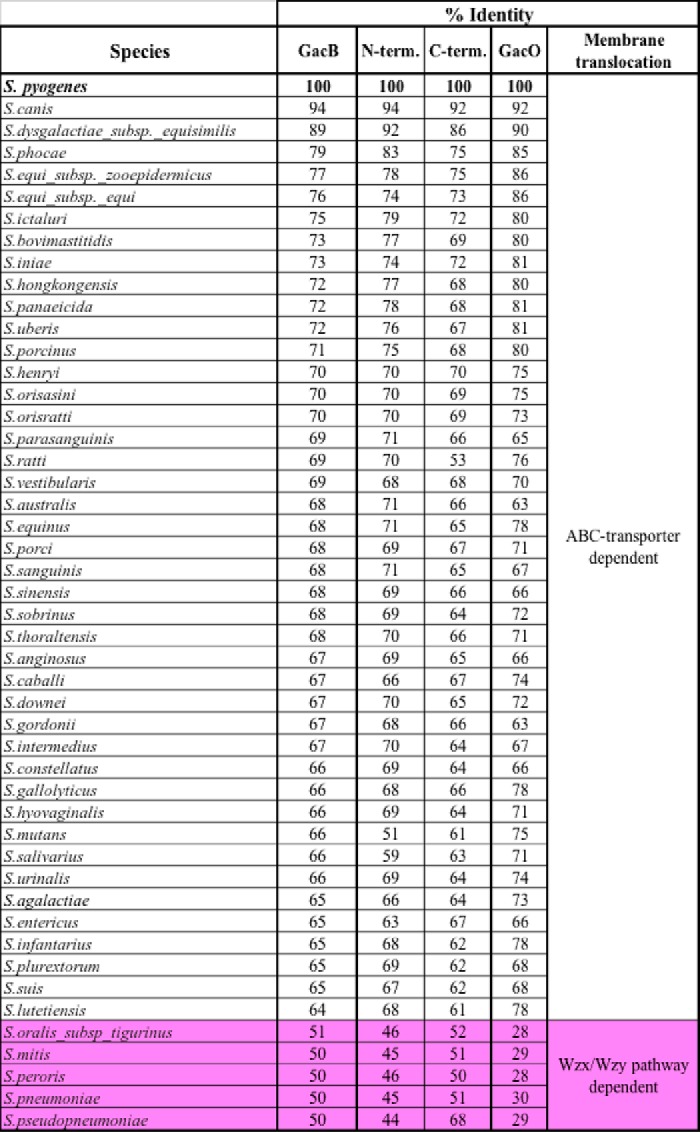
**Sequence conservation in percentage for GacB and GacO homologous enzymes from 48 species of the *Streptococcus* genus** Carbohydrate gene clusters containing either ABC or Wzx/Wzy-encoding genes for carbohydrate translocation and polymerization are marked.

Based on GacB's preliminarily annotation as a nucleotide sugar-dependent rhamnosyltransferase, its genomic location within the GAC cluster, and its similarity to SccB, we selected GacB as the most likely candidate catalyzing the first rhamnosylation step in the GAC biosynthesis process. According to our hypothesis, GacB would attach a single rhamnose residue onto a membrane-bound GlcNAc to form Rha-GlcNAc-PP-Und through an unknown glycosidic linkage.

### GacB is required for the biosynthesis of the GAC RhaPS chain

**Figure 1.**
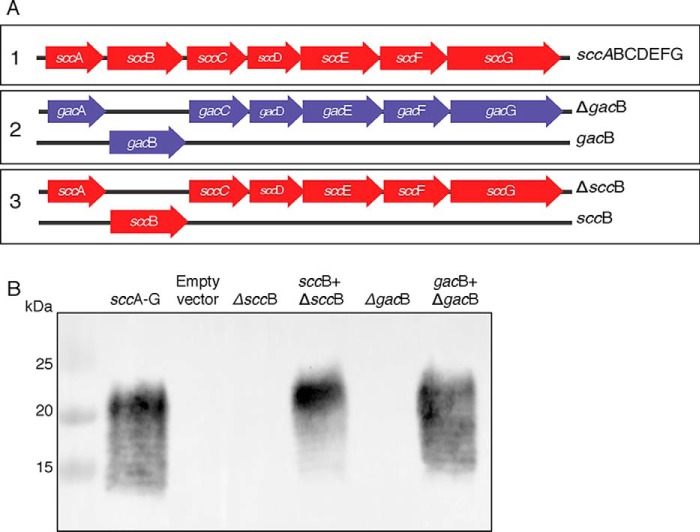
*A*, complementation strategy and map of *S. pyogenes* (*purple*) and *S. mutans* (*red*) genes required to produce the RhaPS polyrhamnose backbone. *S. mutans* cluster: *sccA* (Smu0824), *sccB* (Smu0825), *sccC* (Smu0826), *sccD* (Smu0827), *sccE* (Smu0828), *sccF* (Smu0829), *sccG* (Smu0830). *S. pyogenes* cluster: *gacA* (M5005_Spy_0602), *gacB* (M5005_Spy_0603), *gacC* (M5005_Spy_0604), *gacD* (M5005_Spy_0605), *gacE* (M5005_Spy_0606), *gacF* (M5005_Spy_0607), *gacG* (M5005_Spy_0608). *B*, bacterial complementation assay in *E. coli* CS2775 cells. Immunoblot of a Tricine SDS-PAGE with whole-cell samples probed with anti-Group A carbohydrate antibody.

To further investigate the GacB function and to identify potential catalytic residues, we used *E. coli* as a heterologous expression system to study the GAC RhaPS backbone biosynthesis. Previously, the transformation of *E. coli* cells with the *S. mutans* genes *sccA–G* was sufficient to produce the SCC RhaPS backbone, whereas the absence of *sccB*, encoding the GacB homolog, abrogated the RhaPS backbone biosynthesis (26, 64). Inspired by this approach, we constructed two vectors carrying the homologous genes from *S. pyogenes, gacACDEFG* (*gacA–G*; Δ*gacB*) and *gacB* (Fig. 1*A*).

The RhaPS chain is presumed to be translocated to the outer membrane in *E. coli*, which naturally contains rhamnose attached to the lipopolysaccharides. Thus, to avoid nonspecific binding of the anti-GAC antibody, all transformations were made using an *rfaS*-deficient strain (26). The interruption of the *rfaS* gene impedes the attachment of rhamnose to the LPS on the bacterial outer membrane, rendering a strain that lacks endogenous rhamnose on its surface (26, 62, 65, 66). The role of GacB was investigated using a traditional complementation strategy (Fig. 1).

We investigated the production of RhaPS by *gacA–G* from our complementation approach using immunoblots of total cell lysates (Fig. 1*B*). If the expression of GacBCDEFG is sufficient to produce the RhaPS chain, then we should be able to detect the synthesized RhaPS using a specific anti-GAC antibody. The results showed that *E. coli* cells lacking the *gacA-G* gene cluster (empty vector) did not produce RhaPS (Fig. 1, *lane 2*). Likewise, transformants bearing the Δ*gacB* or Δ*sccB* plasmids lost reactivity with the GAC antibody (Fig. 1, *lanes 3* and *5*). Instead, co-transformation of *sccB* + Δ*sccB* or *gacB* + Δ*gacB* restored the RhaPS production, underlining the essentiality of *sccB* and *gacB* for the biosynthesis of the GAC backbone (Fig. 1, *lanes 4* and *6*).

To investigate whether GacB and SccB are catalyzing the same reaction, we tested the ability of GacB to functionally substitute SccB and *vice versa* by co-transforming Δ*sccB* + *gacB* and Δ*gacB* + *sccB*. In all cases, SccB and GacB were interchangeable (Fig. S2). GacB's predicted initiation codon was different from *S. mutans* SccB, with the latter using TTG instead of ATG (Fig. S2). The use of TTG as initiation codon has been identified as a factor that decreases the efficiency of translation in both *E. coli* and the nematode *Caenorhabditis elegans* (67, 68). Therefore, it is conceivable that this is a strategy used by the cell to limit the expression of the proteins involved in the RhaPS biosynthesis.

Interestingly, the *gacB* homolog in *Streptococcus gallolyticus* subsp. *gallolyticus* (ATCC_43143) is annotated as a shortened, 235-amino-acid-long polypeptide that also contains a TTG codon 153 nucleotides upstream of the annotated GTG start codon. In light of this discovery, a genetic reannotation of *S. mutans*' *sccB* and its homolog in *S. gallolyticus* subsp. *gallolyticus* would be required, as well as a re-evaluation of these species' genome for potentially new or larger gene products that are initiated by a TTG codon.

We decided to test two versions of SccB: one with a TTG as the initiation codon and the other one with an ATG. Both versions rendered an active enzyme that could complement either Δ*scc*B and Δ*gac*B (Fig. S2). Unless stated otherwise, all further work was conducted using *sccB* constructs with the native TTG start codon.

### GacB extends a lipid-linked precursor

We investigated whether GacB is a GT that uses GlcNAc-PP-Und as an acceptor. Through an *in vivo* experiment, we generated radiolabeled lipid-linked saccharides (LLSs), which were isolated from the bacterial membrane and separated via thin-layer chromatography (TLC). Based on the annotation as a rhamnosyltransferase, radiolabeled TDP-β-l-rhamnose would be the preferred sugar donor for GacB. However, this compound is not commercially available; therefore, tritiated glucose was chosen as an alternative. Inside the bacterial cell, glucose is used as a substrate to synthesize a wide array of organic components, including TDP-β-l-rhamnose (69).

**Figure 2.**
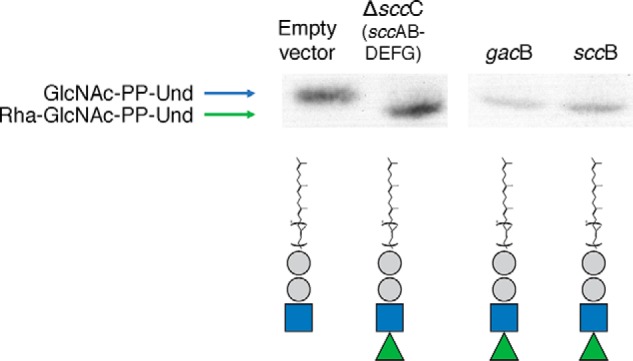
**TLC analysis of radiolabeled LLSs extracted from *E. coli* cells containing the empty vector (negative control) or plasmids encoding *S. mutans* SccAB-DEFG, *S. pyogenes* GacB, or *S. mutans* SccB.** *Below* is the *symbolic representation* of the predicted synthesis products (Und-PP). *Gray circle*, phosphate; *blue square*, GlcNAc; *green triangle*, rhamnose.

We hypothesized that GacB transfers an activated sugar from a (radiolabeled) nucleotide sugar donor to a membrane-bound acceptor monosaccharide-PP-Und (*e.g.* GlcNAc-PP-Und). Therefore, we expect a lower chromatographic mobility (*R_f_*) value of the membrane-bound acceptor, compared with the *R_f_* of the monosaccharide lipid-linked acceptor after running the samples in a TLC plate. As a negative control, we used *E. coli* CS2775 (Δ*rfaS*) transformed with the empty vector. This transformant showed a chromatographic zone consistent with the generation of monosaccharide-PP-Und (Fig. 2, *lane 1*). Upon expression of either the *gacB* or *sccB* genes, we observed the accumulation of a radioactive signal that migrated more slowly on the TLC plate, suggesting a higher molecular mass for these compounds (Fig. 2, *lanes 3* and *4*). The same *R_f_* shift was observed for the *sccAB-DEFG* (Δ*sccC*) construct (Fig. 2, *lane 2*), demonstrating that *sccB* and *gacB* can glycosylate a lipid-linked precursor. Based on the literature, we assume that the upper radiolabeled chromatographic signal corresponds to GlcNAc-PP-Und and the lower one to Rha-GlcNAc-PP-Und (14, 15).

### GacB is a glycosyltransferase transferring rhamnose from TDP-β-l-Rha onto GlcNAc-PP-lipid acceptors

The observed band shift suggested that GacB adds a monosaccharide to a lipid-linked precursor, most likely GlcNAc-PP-Und. We investigated this hypothesis using recombinantly produced and purified GacB WT and amino acid mutants (Fig. S3). We established an *in vitro* assay using the predicted nucleotide sugar donor, TDP-β-l-rhamnose, and a synthetic acceptor substrate. We tested two of these synthetic substrates designed to mimic the native lipid-linked acceptor: CH_3_(CH_2_)_12_-PP-GlcNAc (acceptor 1) and PhO(CH_2_)_11_-PP-GlcNAc (acceptor 2) (Fig. 4*C*). The enzymatic reaction products were purified and characterized using MALDI-MS in positive-ion mode.

**Figure 3.**
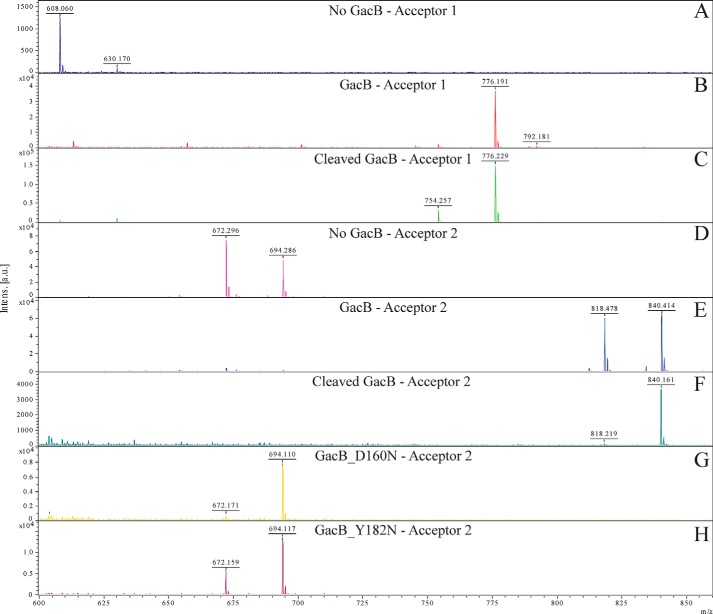
***In vitro* assessment GacB's function detected via MALDI-MS.** Spectra were obtained from the enzymatic reaction sample with the substrate TDP-Rha and acceptor 1 (CH3(CH2)12-PP-GlcNAc) (*A*), acceptor 1 + GacB-GFP (*B*), acceptor 1 + GacB cleaved (no GFP fusion) (*C*), acceptor 2 (PhO(CH2)11-PP-GlcNAc) (*D*), acceptor 2 + GacB-GFP (*E*), acceptor 2 + GacB cleaved (no GFP) (*F*), acceptor 2 + GacB-D160N GFP (*G*), or acceptor 2 + GacB-Y182N-GFP (*H*).

The MALDI-MS spectra of the enzymatic reaction products (Fig. 3) confirmed that GacB catalyzes the addition of one rhamnose residue to both acceptor substrates when incubated with TDP-β-l-Rha (Fig. 3, *B* and *E*). Acceptor 1 possesses a molecular mass of 563 Da and is detected at both *m*/*z* = 608 [M − 1H + 2Na]^+^ and *m*/*z* = 630 [M − 2H + 3Na]^+^ (Fig. 3*A*). GacB-GFP and GacB lacking the GFP tag modified the acceptor, resulting in one predominant peak at *m*/*z* = 776 [M − 2H + 3Na]^+^ (Fig. 3, *B* and *C*). In this spectrum, we can also observe an additional peak of lower intensity at *m*/*z* = 754 [M − 1H + 2Na]^+^, corresponding to the modified acceptor 1 coupled with two Na^+^ ions instead of three Na^+^ ions. In both cases, the products are shifted by *m*/*z* = 146 compared with the unmodified acceptor, which is consistent with the addition of one rhamnose residue via a glycosidic linkage. The same mass shift was observed for the second acceptor; the peaks of the unmodified acceptor 2 (Fig. 3*D*) were detected at *m*/*z* = 672 [M − 1H + 2Na]^+^ and *m*/*z* = 694 [M − 2H + 3Na]^+^, whereas the product peaks emerge at *m*/*z* = 818 [M − 1H + 2Na]^+^ and *m*/*z* = 840 [M − 2H + 3Na]^+^ (Fig. 3, *E* and *F*). We also tested the ability of GacB to catalyze the rhamnosylation of GlcNAc-α-1-P, but the reaction rendered no detectable product (data not shown), suggesting that the enzyme not only interacts with the GlcNAc-α-1-P, but might require the second phosphate and the lipid component to recognize the acceptor substrate.

We further investigated GacB's specificity toward the sugar–nucleotide donor. In particular, we tested whether GacB is selective for thymidine-based nucleotides and tolerates uridine-based nucleotides, such as UDP-Glc, UDP-GlcNAc, and UDP-Rha. As shown before, in the presence of TDP-β-l-Rha, two products consistent with the incorporation of rhamnose residue plus either two or three sodium cations were observed in the spectrum (Fig. S4*A*). In contrast, no product peaks were observed with the α-d-configured substrates UDP-α-d-Glc or UDP-α-d-GlcNAc (Fig. S4, *B* and *C*), whereas residual activity was detected for UDP-β-l-Rha (Fig. S4*D*). These data demonstrate that GacB does not tolerate α-d–configured nucleotide sugars. Furthermore, GacB has specificity toward the deoxythymidine moiety (TDP-Rha) or requires binding of the thymine methyl group.

Finally, we assessed metal ion dependence *in vitro*. Compared with the control reaction (Fig. S5*B*), we noticed no significant differences in the rhamnosylation activity of the enzyme when GacB was supplemented with MgCl_2_, MnCl_2_, or EDTA as a metal chelator (Fig. S5, *C–E*), indicating that GacB does not require a divalent metal ion for its activity. These data are in agreement with the lack of a conserved D*X*D motif found in metal-dependent GTs (70, 71).

Together, these data confirmed our previous conclusions drawn from the LLSs radiolabeled assay (Fig. 2). These results provide the first *in vitro* evidence revealing that GacB is a metal-independent rhamnosyltransferase that catalyzes the initiation step in the GAC RhaPS backbone biosynthesis by transferring single rhamnose to GlcNAc-PP-Und using TDP-β-l-Rha as the exclusive activated nucleotide sugar donor.

### Investigation of GacB's catalytic residues

There are no available crystal structures of proteins with a high degree of identity to GacB. Therefore, we constructed a GacB structural model based on two enzymes that belong to the GT4 family of GTs: *Bacillus anthracis*' *Ba*BshA (PDB entry 3MBO) (72) and *Corynebacterium glutamicum*'s MshA (PDB entry 3C4V) (57). Both proteins have low amino acid sequence identity compared with GacB, making the identification of potential catalytic residues to mutate in GacB very challenging. *Ba*BshA shares only 15% identity in only 64 of 424 amino acids. MshA is an equally distant “homologous” GT that shares 16% identical residues in a sequence stretch of only 71 residues of 446. Based on the scarce information provided by the structural models and the multiple-sequence alignment described in detail below, we mutated several residues that are highly conserved in over 40 pathogenic streptococci species (Fig. S7).

Our *in vitro E. coli* system is the first one that enables the study of GacB mutant proteins, allowing the identification of those mutants that abrogate or reduce the production of RhaPS backbone. Conducting this in *S. pyogenes* is not possible, as *gacB* is essential (7, 26). We used the information available from the GT models mentioned above and the sequence alignment of multiple streptococci to select residues that might be involved in substrate binding, which tends to be conserved among GT. Through *in situ* mutagenesis, we constructed nine recombinant versions of GacB containing the following amino acid substitutions: D126A, D126N, E222A, E222Q, D160A, D160N, Y182A, Y182F, and K131R. The latter mutation was included as a negative control because it is a conserved predicted surface residue that presumably is not engaged in the catalytic activity or could inactivate the enzyme otherwise.

Our results show that substitution of Asp-160 with an asparagine (Fig. S6, *lane 5*) led to a drastic reduction in the production of the RhaPS chain, whereas an alanine residue did not cause such a significant effect. This suggests that the Asp-160 carboxyl group might be required for catalysis, which potentially can be replaced in the alanine mutant by a water molecule. A more severe effect was observed with mutations of Tyr-182. The alanine substitution of Tyr-182 (Y182A) impeded the RhaPS backbone biosynthesis significantly, whereas Y182F completely inactivated GacB (Fig. S6, *lanes 9* and *10*), suggesting an essential role for the Tyr-182 hydroxyl group in GacB's enzymatic activity.

We further investigated the mutants D160N and Y182F in an *in vitro* assay using recombinantly expressed and purified GacB-GFP fusions (Fig. S3). The MALDI-MS analysis of the reaction products from GacB-D160N-GFP and GacB-Y182F-GFP revealed that both mutants lacked an enzymatic activity *in vitro* (Fig. 3, *G* and *H*). These results support the hypothesis that the residues Asp-160 and Tyr-182 play a role in substrate binding or catalysis.

Finally, we created three truncated versions of GacB at the N-terminal end as an attempt to determine whether the enzyme remains active in the absence of the residues predicted to be associated with the membrane. Our results showed that truncations of the first 22 (GacB_23–385_), 75 (GacB_76–385_), and 118 residues (GacB_119–385_) led to inactivation of the enzyme when assessed through the complementation assay (Fig. S6, *lanes 11–13*). Their inability to complement Δ*gacB* suggest that the N-terminal domain is required for activity and supports the hypothesis that GacB is a membrane-associated rhamnosyltransferase.

### GacB is a retaining β-1,4-rhamnosyltransferase

The current gene annotation suggests that GacB is an inverting α-1,2-rhamnosyltransferase (7, 14). This annotation is incompatible with the acceptor sugar GlcNAc because its carbon at position C2 is already decorated with the *N*-acetyl group. Therefore, GacB can only transfer the rhamnose onto the available hydroxyl groups on C3, C4, or C6. The GAC backbone is composed of disaccharide repeating units of rhamnose connected via α-1,3-α-1,2 glycosidic bonds (15, 18, 85), suggesting that GacB could be using a retaining mechanism of action, synthesizing an α-1,3, α-1,4, or α-1,6 glycosidic linkage. According to the CAZy database, the GacB sequence is classified as a GT4 family member, which are classified as retaining GTs (73). If that classification is correct for GacB, the stereochemical configuration at the anomeric center of the sugar donor, TDP-β-l-rhamnose, should be retained in the final product (56, 64, 74).

**Figure 4.**
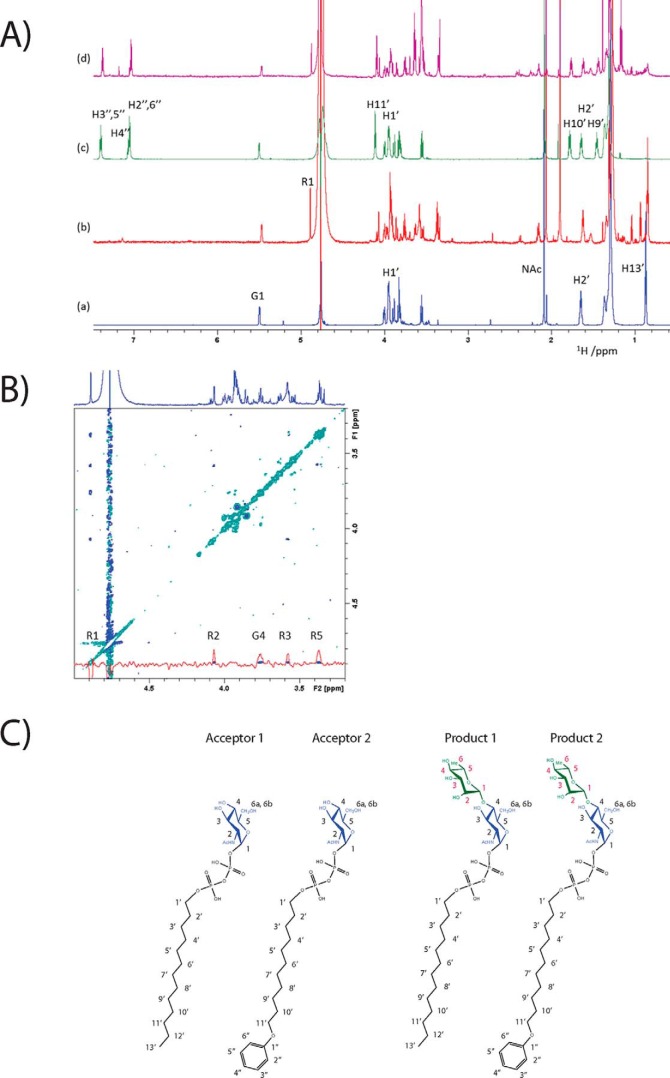
*A*, 800-MHz ^1^H NMR spectra of acceptor substrate 1 (*a*), product 1 (*b*), acceptor substrate 2 (*c*), and product 2 (*d*). *B*, partial 2D ROESY spectrum of product 1 showing the correlations between the H1 of β-l-Rha and the protons of rhamnose (*R*) and protons of GlcNAc (*G*). The F2 cross-section through H1 of Rha is shown in *red. C*, chemical structures with proton numbering.

To elucidate whether GacB is an inverting or a retaining rhamnosyltransferase, we conducted NMR spectroscopy on the purified reaction products 1 and 2. ^1^H NMR spectra were collected at 800 MHz to both establish the structural integrity of acceptors 1 and 2 (Fig. 4*A*) and determine the chemical structure of their products after the enzymatic reaction (products 1 and 2). The NMR parameters were determined through one- and two-dimensional (1D and 2D) and 2D total correlation spectroscopy (TOCSY) experiments (Fig. 4*B*); their chemical shifts are summarized in Table 2. For both acceptors, the anomeric proton of the α-d-GlcNAc residue appeared as a doublet of doublets with 3J(H1,H2) = 3.4 Hz and 3J(H1,P) = 7.2 Hz. Proton H2 of α-d-GlcNAc was also split by a 3J(H2,P) = 2.4 Hz coupling with P. A 2D 1H, ^31^P HMQC spectrum (data not shown) revealed a correlation of both of these H-1′ protons with P at −13.5 ppm. Another correlation appeared between the ^31^P at −10.6 ppm and protons of the adjacent CH_2_ groups of the alkyl chain, confirming the integrity of the acceptor substrate. For acceptor 2, a typical pattern of signals of monosubstituted benzene with integral intensities of 2:2:1 was observed.

**Table 2:**
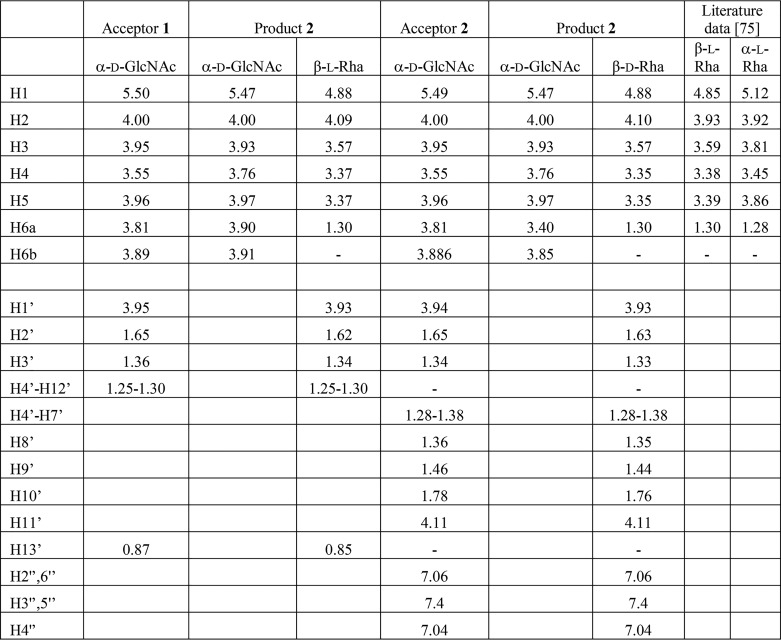
**^1^H chemical shifts (ppm) of acceptor substrates and products 1 and 2**

The addition of rhamnose to both acceptor substrates was accompanied by the appearance of a characteristic signal in the anomeric region of the spectrum (4.88 ppm, H1) next to the water signal. The anomeric configuration of this monosaccharide was established in several ways. The measured ^3^*J*(H1,H2) coupling constant of 1.0 Hz indicated a β-l-configuration (1.1 and 1.8 Hz reported) (75) for β-l- and α-l-Rha, respectively). Furthermore, we complemented our studies by investigating the product rotating-frame NOE (ROESY) spectrum (Fig. 4*B*). Importantly, the ROESY spectrum showed spatial proximity of H1 of rhamnose with four other protons. Among these were H2, H3, and H5 protons of rhamnose, the latter two confirming a 1,3-diaxial arrangement between H1, H3, and H5 that is indicative of a β-l-Rha configuration. Finally, a comparison of ^1^H chemical shifts of rhamnose with those of α-l- and β-l-rhamnopyranose (Fig. 4*C*) showed a good agreement with those of β-l-rhamnose but not α-l-rhamnose (75) (Table 2), thus further confirming the anomeric configuration of this ring. The forth ROESY cross-peak of H1 of rhamnose was with H4 of GlcNAc, revealing the presence of a (1→4) linkage between the two monosaccharides. This observation was further supported by a comparison of GlcNAc ^1^H chemical shifts of acceptor substrates and products. Here, an increased chemical shift (+0.21 ppm) was observed for H4 upon glycosylation, whereas the average of the absolute values of the differences between the chemical shifts of the other corresponding protons of GlcNAc was 0.03 ppm. As expected, the signals of the alkyl and aryl radicals practically did not change in the respective acceptor-product pairs.

In conclusion, ^1^H NMR spectroscopy revealed the formation of a β-l-Rha (1→4) d-GlcNAc moiety and the integrity of the product. Other enzymes from Gram-negative and Gram-positive bacteria that are involved in polysaccharide biosynthesis also use lipid-linked GlcNAc as the initial acceptor sugar, whereas their nucleotide-sugar donor is either TDP-l- or GDP-d-rhamnose. However, their respective reaction products are α-1,3 or α-1,4 glycosidic bonds (76–81). Product analysis, therefore, undoubtedly reveals that GacB is the first retaining GT that synthesizes a novel d-GlcNAc-β-1,4-l-rhamnose disaccharide using TDP-β-l-rhamnose as the exclusive nucleotide sugar donor.

### Group A, B, C, and G Streptococcus share a common RhaPS initiation step

**Figure 5.**
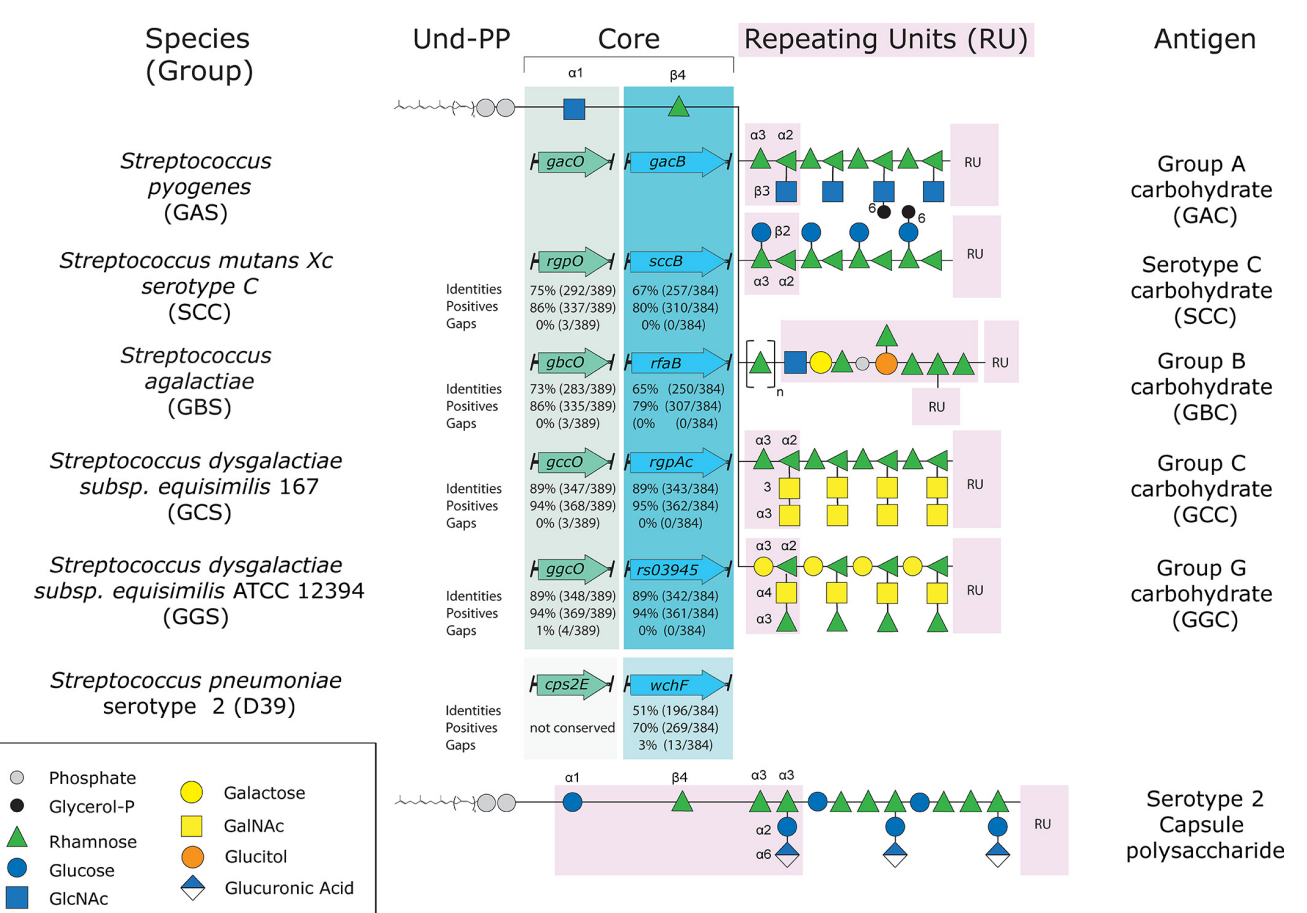
***Schematic representation* of the RhaPS initiation within different *Streptococcus species* compared with the capsule polysaccharide in *S. pneumoniae*.** RhaPS biosynthesis is initiated on Und-P by GacO (*green background*), followed by the action of GacB (*turquoise*), generating the conserved core structure Und-PP-GlcNAc-Rha. Percentage of the amino acid sequence identity, positive amino acids, and gaps within the sequence compared with GacO or GacB are given *below* each homolog: *S. mutans* Xc serotype C SccB, *S. agalactiae* (GBS) RfaB, *Streptococcus dysgalactiae* subsp. *equisimilis* 167 (GCS) RgpAc, and *S. dysgalactiae* subsp. *equisimilis* ATCC 12394 (GGS) Rs03945. The specific carbohydrate composition extending the lipid-linked core structure of each group is depicted on the *right*. Carbohydrate repeat units (*RU*) are highlighted (*light pink background*), and *symbolic representations* of the sugars are shown in the *key*.

In addition to *S. mutans* SccB, GacB homologs with a high degree of sequence identity are found in other streptococcal species of clinical importance, such as the *Streptococcus* species from Group B (GBS), Group C (GCS), and Group G (GGS). All homologous enzymes are situated in the corresponding gene clusters encoding the biosynthesis of their Lancefield antigens (*i.e.* the Group B, C, and G carbohydrate) (15). The homologous gene products share 67, 89, and 89% amino acid identity to GacB, respectively (Table 1 and Fig. 5). With varying degrees of evidence, depending on the species, there is a general understanding of the chemical structure of the RhaPS of these streptococci (15). The currently accepted structures for GAC, GBC, GCC, GGC, and SCC are summarized in Fig. 5 (14–16, 23, 82, 83). Remarkably, none of the investigations that led to the understanding of the surface carbohydrate structures include data describing the mechanism of action of the enzymes involved in the priming step of each RhaPS biosynthesis.

**Figure 6.**
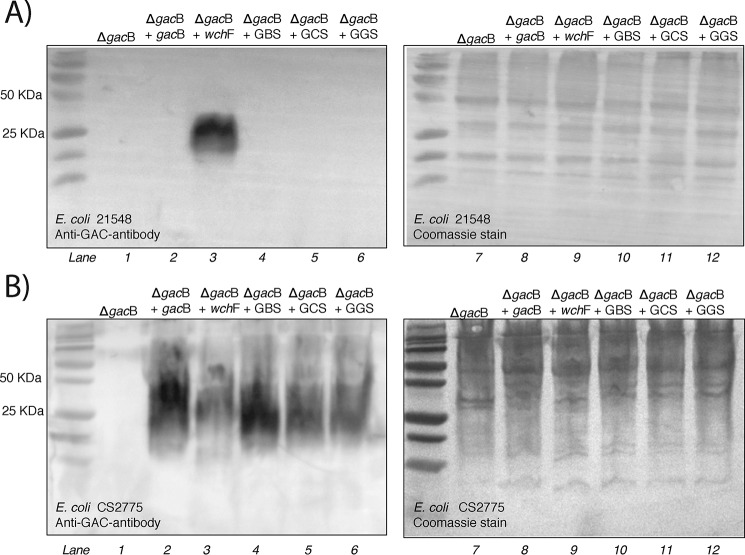
**Bacterial complementation assay in *E. coli* 21548 (*A*) and *E. coli* CS2775 (*B*).** Shown is immunoblotting of Tricine SDS-PAGE with whole-cell samples probed with anti-Group A carbohydrate antibody and membranes after Coomassie stain.

Based on the high-sequence identity to GacB, we hypothesized that the carbohydrate biosynthesis of the Group A, Group B, Group C, and Group G *Streptococcus* possesses a conserved initiation step, in which the first rhamnose residue is transferred onto the lipid-linked acceptor forming α-d-GlcNAc-β-1,4-l-rhamnose-PP-Und. We tested the ability of the homologs from GBS, GCS, and GGS (GbsB, GcsB, and GgsB, respectively) to functionally substitute GacB in the production of the RhaPS chain (Fig. 6). Our results show that all homologous proteins were able to restore the RhaPS backbone when their genes were co-expressed with the Δ*gacB* expression plasmid, suggesting that these enzymes can perform the same enzymatic reaction.

We showed that GacB requires GlcNAc-PP-Und as acceptor (Fig. 6), but it is possible that the enzymes from GBS, GCS, and GGS use a different lipid-linked acceptor substrate, such as Glc-PP-Und. Thus, to determine whether the GacB homologs require GlcNAc-PP-Und as lipid acceptor, we conducted the complementation assay using *E. coli* Δ*wecA* cells, which lack GlcNAc-PP-Und (62). As a positive control, we identified *Streptococcus pneumoniae* WchF, a α-d-Glc-β-1,4-l-rhamnosyltransferase that uses Glc-PP-Und exclusively as substrate (84). As expected, GacB was unable to restore the RhaPS chain when co-transformed with the Δ*gacB* vector in the absence of the GlcNAc-PP-Und (Fig. 6*A*, *lane 2*). Importantly, the GacB homologs from GBS, GCS, and GGS also failed to produce the RhaPS backbone (Fig. 6*A*, *lanes 4–6*) but could replace GacB function in the Δ*rfaS* strain (Fig. 6*B*). Only WchF, which uses a Glc-PP-Und acceptor for the transfer of a rhamnose residue (83), restored the RhaPS biosynthesis in the absence of GlcNAc-PP-Und (Fig. 6*A*, *lane 3*). Combined with the data from our *in vitro* enzymatic reactions, these results strongly suggest that the GacB homologs from GBS, GCS, and GGS are also α-d-GlcNAc-β-1,4-l-rhamnosyltransferase that require GlcNAc-PP-Und as membrane-bound acceptor.

### d-GlcNAc- or d-Glc-1,4-β-l-rhamnosyl-transferases define translocation mechanism in streptococcal pathogens

**Figure 7.**
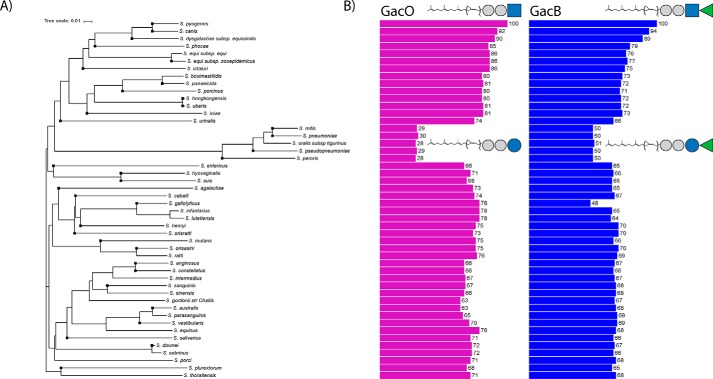
*A*, phylogenetic tree of GacB homolog sequences identified from 48 partially or completely sequenced streptococcal pathogens. The tree was constructed based on multiple-sequence alignment of GacB homologs using the default neighbor-joining clustering method of Clustal Omega. The tree was plotted using iTOL online tool. *Black squares* at the *branches* indicate species with fully sequenced genomes. *B*, *bar charts* associated with each node indicate the amino acid identity (percentage) of the respective homologs to GacO (magenta) or GacB (*blue*) from *S. pyogenes*.

*S. pneumoniae* serotype 2 *wchF* encodes a α-d-Glc-β-1,4-l-rhamnosyltransferase that requires Glc-PP-Und as acceptor (84). The WchF enzyme conducts the first committed step for Wzx/Wzy-dependent capsule biosynthesis. Interestingly, it shares only 51% amino acid identity to GacB, compared with 67–89% for the homologous enzymes from GBS, GCS, GGS, and *S. mutans* (Table 1). Toward a better understanding of the conservation of GacB in the *Streptococcus* genus, we extended our bioinformatics analysis to search for other strains that harbor *gacB* homologous genes. We found 48 human/veterinary pathogenic *Streptococcus* species with a single GacB homolog, sharing 50–94% amino acid sequence identity (Table 1 and Fig. 7). Interestingly, only five of our 48 identified species showed a percentage identity equal to or lower than 51% (*Streptococcus mitis*, *S. pneumoniae*, *Streptococcus oralis* subsp. *tigurinus*, *S. peroris*, and *Streptococcus pseudopneumoniae*), whereas all other encoded proteins presented more than 65% homology to GacB. For simplicity, we will refer to the five *Streptococcus* strains with low amino acid identity as the “low-identity” subgroup, and the rest of the species as the “high-identity” subgroup. The sequence analysis paired with the complementation assay led us to hypothesize that all GacB homologs encompassed in the high-identity subgroup possess d-GlcNAc-β-1,4-l-rhamnosyltransferase activity. In contrast, the low-identity subgroup contains *S. pneumoniae* WchF, a known d-Glc-β-1,4-l-rhamnosyltransferase (84). Interestingly, all five members of the low-identity subgroup exhibit very high sequence identity (>90%) when compared with WchF, therefore strongly suggesting that they are WchF homologs that also synthesize Rha-β-1,4-Glc-PP-Und.

GacO from *S. pyogenes*, the WecA homolog, was shown to be responsible for the biosynthesis of the GlcNAc-PP-Und (14, 15), the substrate for GacB. We therefore hypothesized that the low- and high-identity subgroups utilize different substrates and therefore investigated whether a similar discrepancy should be observed when comparing the sequence identity of the GacO homologs. Strikingly, within the 48 pathogenic streptococci genomes (Table 1 and Fig. S7), we found that all strains from the high-identity subgroup share a GacO homolog with 63–92% sequence identity. Importantly, any genome from the low-identity subgroup contains a gene product with ≤30% sequence identity to GacO. This subgroup contains gene products that have high homology to *S. pneumoniae* Cps2E, which transfers Glc-1-P to P-Und, to generate Glc-PP-Und (51, 84). *S. mitis*, *S. oralis* subsp. *tigurinus*, *S. peroris*, and *S. pseudopneumoniae* homologs share 98% sequence identity to Cps2E. Thus, we propose that their capsule polysaccharides are also built on Glc-PP-Und.

The degree of phylogenetic conservation of GacB in the *Streptococcus* genus highlights the importance of this gene, and perhaps of the entire RhaPS gene cluster, for survival and pathogenesis of streptococcal pathogens. Overall, these results lead us to propose that those streptococcal species that have GacB homologs with a high degree of identity (>65%) are d-GlcNAc-β-1,4-l- rhamnosyltransferases that catalyze the first committed step in the biosynthesis of their surface RhaPS by transferring rhamnose from TDP-β-l-rhamnose to the membrane-bound GlcNAc-PP-Und. In contrast, we postulate that the species encompassed in the low-identity subgroup, following the function of *S. pneumoniae* serotype 2 WchF, contain a rhamnosyltransferase that acts on lipid-linked Glc-PP-Und.

It is worth noticing that the capsule biosynthesis in *S. pneumoniae* and other Gram-positive bacteria occurs via the Wzx/Wzy-dependent pathway, and the polymerization of the glycan occurs *after* the export of the repeating unit by Wzy (84–86). Importantly, ∼75% of all *S. pneumoniae* capsular polysaccharides are synthesized onto the reducing end of lipid-linked Glc, and strikingly, the elongation of the repeat unit occurs on that very same glucose (87, 88). This information strongly suggests that a reducing end GlcNAc is not compatible with the elongation step via the polymerase Wzy. The Wzx translocation mechanism is commonly used for the synthesis of several surface carbohydrates of lactic acid bacteria, as well as for the production of the lipopolysaccharide and capsules in Gram-negative species (78–80). In contrast, the elongation step of the GAC biosynthesis pathway is proposed to take place in the cytoplasm *before* the translocation via the hypothesized ABC transporter. The species encompassed within the low-identity subgroup all contain Wzx/Wzy genes downstream of the WchF homolog, whereas the high-identity subgroups share the same type of ABC transport genes with their respective surface RhaPS gene clusters (Table 1).

### GacB's N-terminal domain encodes specificity for the GlcNAc acceptor

We decided to further investigate the role of the N- and C-terminal domains in acceptor substrate recognition. GTs show a high degree of specificity toward both the sugar acceptor and the nucleotide-activated sugar donor (89). Although the majority of GTs possess either a GT-A or GT-B type structure fold, the direct function of the GTs cannot be predicted, as they do not tend to share conserved residues associated with the enzymatic activity or have a common mechanism of action (64, 89). Nevertheless, it has been observed that the N-terminal domain of GT-B folded GTs is responsible for the acceptor substrate recognition, whereas the C-terminal domain is involved in sugar–nucleotide donor binding (90). Despite the poor sequence identity to known GT4 protein sequences, we trusted our bioinformatic analysis indicating that GacB has structural similarity to the GT4 family, a group of retaining GTs that have a GT-B fold. We have revealed that GacB is a retaining GT, and our sequence analysis revealed that the protein has two distinct domains. We investigated whether this observation correlates to a lower sequence percentage identity in the N-terminal domain than in the C-terminal domain when compared with WchF. If this were the case, then these differences should also extend to the other species encompassed in the high-identity subgroup.

We performed multiple-sequence alignment of the GacB homologs from all 48 streptococcal pathogens to identify the most variable and conserved regions in the protein sequence. We observed a higher discrepancy between the high-identity and the low-identity subgroups in their N-terminal domains (Table 1 and Fig. S7). More precisely, a low sequence conservation region is identifiable between the GacB amino acid residues 40 and 80, suggesting that this section of the domain is either involved in the GlcNAc acceptor sugar recognition or essential protein–protein interactions.

We knew from our previous experiment that GacB could not initiate the RhaPS biosynthesis on a *wecA* deletion background (Fig. 6*A*, *lane 2*). Based on this information and to identify residues involved in sugar acceptor recognition, we introduced mutations in the GacB amino acid sequence. The goal was to salvage the RhaPS initiation step in a *wecA*-deficient *E. coli* strain in which GacB mutants recognize a lipid-linked sugar acceptor other than GlcNAc-PP-Und. *E. coli* contains the *wcaJ* gene that encodes the enzyme that synthesizes Glc-PP-Und.

**Figure 8.**
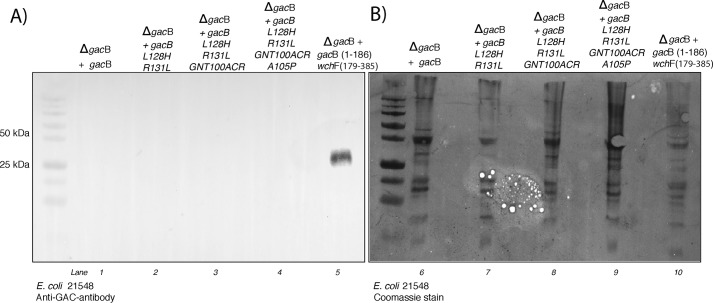
**Bacterial complementation assay in *E. coli* 21548 cells, lacking the gene *wecA* and subsequently lacking GlcNAc-PP-Und as an acceptor for lipid-linked carbohydrate biosynthesis.** *A*, immunoblot of whole-cell samples probed with anti-Group A carbohydrate antibody and Coomassie staining (*right*) (*B*).

Therefore, we investigated a structural model based on the GacB homolog from *Bacillus anthracis*, BaBshA (PDB entry 3MBO), which suggested that residues Leu-128 and Arg-131 and Gly-100, Asn-101, Thr-102 (GNT100) may potentially be involved in sugar acceptor recognition. We mutated these residues to mimic those found in WchF. Complementation assays using GacB L128H_R131L failed to complement Δ*gacB* in a Δ*wecA* background (Fig. 8, *lane 2*). Following a sequential approach, we modified GacB primary sequence by introducing additional amino acid substitutions that corresponded to those found in WchF: L128H_R131L_GNT100ARC and L128H_R131L_GNT100ARC_A105P. None of these mutant proteins were able to initiate the rhamnose chain on Und-PP-Glc and, thus, did not restore GacB's activity. Finally, we replaced the first 178 residues of GacB with the corresponding WchF amino acids (residues 1–186). When expressed in a *wecA* deletion background, this WchF-GacB chimera was able to synthesize the RhaPS backbone on the exclusive acceptor substrate Glc-PP-Und (Fig. 8, *lane 5*).

In light of our findings, we postulate that the C-terminal domain of GacB is involved in the GT reaction, whereas the N-terminal domain, which includes the uncharacterized DUF1972 domain, participates in lipid-linked substrate recognition and binding. A distinction in domain functionality has been suggested for other GTs of the GT4 family. For instance, in MshA, a GT4 in *C. glutamicum*, the donor substrate binding occurs at the C-terminal domain through the residues located at the β_12_/α_10_ loop, whereas the N-terminal domain is involved in acceptor binding (57). Likewise, biochemical and structural studies on WsaF, a rhamnosyltransferase involved in elongating the polysaccharide's backbone chain in the Gram-positive bacterium *Geobacillus stearothermophilus* NRS 2004/3a, indicate that the acceptor-binding region is located at the N-terminal domain, whereas the C-terminal domain is associated with sugar donor transfer (85, 86). Interestingly, WsaF's C-terminal end is involved in TDP-Rha binding and contains an E*X*_7_E motif, also found among its homologs and other putative rhamnosyltransferases. Remarkably, all GacB streptococcal homologs contain a similar G*X*_7_E motif that may be involved in the GT activity. This sequence contains neither the proline-rich motif nor a conserved tyrosine (Tyr-329) that is suggested to be involved in the rhamnose binding described for WsaF (85) (Fig. 8).

Furthermore, GacB-related homologs have a fully conserved GGTNPGLLE sequence, whereas the *S. pneumoniae* WchF-like subgroup has a GGTNPSLLE sequence, suggesting that yet another mechanism may be used to bind and select for TDP-Rha in streptococcal Lancefield species. In terms of the mechanism of action, kinetic and structural data collected from other retaining rhamnosyltransferases of the GT-B family suggest that the stereochemical outcome is a result of a double-displacement mechanism facilitated by interdomain flexibility (66, 72, 76, 78, 87–90). However, further biochemical and structural work is required to pinpoint the catalytic residues in GacB and its homologs.

Altogether, these results, although insufficient to pinpoint the individual residues that recognize the lipid sugar acceptor, demonstrate that GacB belongs to the GT-B superfamily and has a high substrate selectivity toward the GlcNAc acceptor and TDP-β-l-rhamnose as the nucleotide sugar donor. Importantly, our data strongly suggest that the acceptor recognition takes places at the N-terminal domain, opening the possibility of assigning a role in acceptor binding to the domain of unknown function DUF1972 as the GlcNAc-PP–binding site.

## Conclusions

In recent years, the biosynthesis of GAC, a major structural component of the cell wall in *S. pyogenes* (14, 15), has received increased attention. The work of van der Beek *et al.* in 2015 (24) shed light on the role of GacA in the production of the nucleotide sugar TDP-β-l-rhamnose, which is used to build the GAC backbone chain (7). Collectively, the research of Rush *et al.*(14) and Edgar *et al.* (18) revealed the role of GacHIJKL, the enzymes involved in chain modification after the translocation step. Despite this progress, the production of the RhaPS backbone, which involves the initiation and elongation stages of the GAC biosynthesis, remained unexplored. Our work unveils the function of GacB, the first GT of this pathway, which conducts a conserved priming step in the RhaPS biosynthesis.

Several research groups have shown that studying the GAC cluster directly in *S. pyogenes*, including the generation of conditional knockouts, constitutes a real technical challenge (7, 24). The genes encoding GacB, as well as GacA, GacC–G, and GacL, were indispensable for *S. pyogenes* survival in transposon mutagenesis experiments (7, 10, 25).

It remains unknown whether the GAC is essential for Group A *Streptococcus* survival or if the lethal phenotype observed after deletion of genes of the gene cluster is a result of the accumulation of GlcNAc-PP-Und, GlcNAc-P-Und, or any other intermediate LLS. For instance, the accumulation of the lipid-linked precursor has been observed after the deletion of *gacABC*, *gacG*, and *gacL* homologous genes involved in *S. pneumoniae* serotype 2 capsule biosynthesis and in *Bacillus subtilis* and *Staphylococcus aureus* wall teichoic acid biosynthesis (22, 73, 84). Importantly, until today, no gene has been identified in *Streptococcus* species that could convert/recycle these lipid-linked intermediates, as has been described for other bacteria (99).

In *B. subtilis* and *S. aureus*, secondary deletions of the early-acting gene, such as the one involved in GlcNAc-PP-Und biosynthesis, *tagO/tarO* (73), reverse the lethal phenotype observed as a consequence of interrupting the polymerization of these secondary cell wall structures. Under those circumstances, the genes involved in wall teichoic acid polymerization are no longer essential, albeit their deletion leads to cells with compromised fitness, and this phenomenon could also be the case for the GAC biosynthesis pathway. Importantly, disruption of the *gacO* gene has been shown to cause cell death (25). However, van Sorge *et al.* (7) showed that low concentrations of tunicamycin (0.025%), a specific inhibitor of undecaprenyl-phosphate GlcNAc-1-phosphate transferase (WecA/GacO) producing GlcNAc-PP-Und, lead to viable *S. pyogenes* cells with no RhaPS in their cell walls (25). Therefore, it would be interesting to investigate whether the *gac* gene deletions mentioned earlier cause the same lethal phenotype in tunicamycin-treated *S. pyogenes* strains.

We have demonstrated that GacB is a functional homolog of SccB, the proposed first rhamnosyltransferase in the RhaPS synthesis pathway of *S. mutans* serotype c (26). Previous research on *S. mutans* and *in silico* studies of the Group B *Streptococcus* polysaccharide biosynthesis clusters showed that GacB homologs are likely to be rhamnosyltransferases, but no biochemical evidence was ever provided to support these hypotheses (26, 82, 91). Here, we provide the first biochemical confirmation that GacB is a β-1,4-l-rhamnosyltransferase, in opposition to its preliminary annotation (7, 14, 15). We also reveal that GacB does not require divalent cations for its activity, which is consistent with the absence of a D*X*D motif in its primary sequence (70, 89) (Fig. S5).

The combined results presented here encourage GacB's inclusion in the GT4 subfamily, although the enzymes of this CAZy family are currently limited to those who utilize α-configured nucleotide sugars. Furthermore, the data presented in this work support the hypothesis that the N-terminal domain is responsible for the binding of the lipid-linked substrate as well as the association to the bacterial membrane in the cytosol. We speculate that the domain of unknown function DUF1972 is fulfilling this role.

We observed that mutations in the highly conserved residues Asp-160 and Tyr-182 abrogated GacB's priming activity in both the *in vivo* complementation and the *in vitro* enzymatic activity assays (Fig. 3 and Fig. S6). In the absence of a crystal structure or one from closely related homologs, we can only speculate on the role of these residues based on the existing literature of other GT-B retaining GT. Tyrosine residues have been described in the literature as essential active-site residues for the catalytic activity of several glycosyltransferases. Their proposed roles are diverse and range from acting as a catalytic base, where the Tyr side chain can activate the acceptor hydroxyl group (92), to constituting nucleotide sugar ribose-binding residues (93). In this context, we hypothesize that the hydroxyl group of Tyr-182 might fulfill one of the two proposed roles in the GAC initiation process. In the absence of a crystal structure, or a suitable homologous structure, we cannot reveal the mechanistic details of GacB and the role of this conserved Tyr-182.

Another exciting outcome of this research has been the finding that the reaction catalyzed by GacB, the formation of Rha-β-1,4-GlcNAc-PP-Und, is highly conserved in the *Streptococcus* genus. The bacterial complementation assays and the bioinformatics analysis lead us to hypothesize that this initiation step is shared by all streptococcal pathogens expressing a RhaPS surface carbohydrate (Table 1 and Figs. 6 and 7), including but not limited to species from the Lancefield Groups A, B, C, D, E, F, G, H, K, L, M, N, O, P, and R.

Overall, this work unveils the function of GacB, the first metal-independent, retaining, and nonprocessive α-d-GlcNAc-β-1,4-l-rhamnosyltransferase reported, and sheds light on the first committed step of the RhaPS biosynthesis in Group B, Group C, and Group G *Streptococcus*.

Importantly, GacB's essentiality and the lethal phenotype of GAC-depleted strains, in addition to the high-sequence conservation observed for GacB homologs from several streptococcal pathogens, may have significant implications for the future development of multitarget drugs in polypharmacology, because humans lack both TDP-Rha and related glycosyltransferases. Finally, our insights regarding the ability to synthesize streptococcal RhaPS in a recombinant expression system, namely *E. coli*, onto different lipid-linked acceptor sugars by substituting the enzyme GacB (or its homologs) provide exciting new opportunities for glycoconjugate vaccine development.

## Experimental procedures

### Bioinformatics analysis

Alignments of protein sequences were performed using NCBI Blast Global align (https://blast.ncbi.nlm.nih.gov/Blast.cgi) and ClustalOmega (https://www.ebi.ac.uk/Tools/msa/clustalo/)^5^ (94). Molecular weight predictions were obtained using the ProtParam tool at the Expasy server (http://www.expasy.org/) (101).^5^ Protein topological predictions were generated using both SpOctopus (http://octopus.cbr.su.se/)^5^ (102) and the TMHMM algorithms (www.cbs.dtu.dk/services/TMHMM/).^5^

Secondary structure predictions were generated using either Phyre2 (http://www.sbg.bio.ic.ac.uk/phyre2/html/page.cgi?id=index)^5^ (103) or RaptorX (raptorx.uchicago.edu)^5^ and visualized using PyMOL (educational version 1.8, Schrödinger, LLC). The Carbohydrate-Active enZymes database (CAZy) (http://www.cazy.org/)^5^ (73) was examined to obtain information about the classification and characterization of carbohydrate active enzymes. Phylogeny relationships were established using Clustal Omega, Clustal X, and iTOL (95).

### Bacterial strains and growth conditions

All bacterial strains used in this study are listed in Table 3. *E. coli* strains DH5α and MC1061 were used indistinctively as host strains for the propagation of recombinant plasmids and plasmid integration. *E. coli* CS2775, a strain sourced from Prof. Rodney Welsh that lacks the Rha modification on the lipopolysaccharide, was used as the host strain to evaluate the production of RhaPS. *E. coli* 21548 is a Und-PP-GlcNAc–deficient strain that contains a *wecA* deletion, serving as a negative control for the production of RhaPS. The strain was sourced from Dr. Jeffrey Rush. *E. coli* strain C43 (DE3) was used for the production of recombinant protein. All *E. coli* strains were grown in lysogeny broth media. Unless otherwise indicated, all bacterial cultures were incubated at 37 °C in a shaking incubator at 200 rpm. Where necessary, media were supplemented with one or more antibiotics to the following final concentration: carbenicillin (Amp) at 100 μg/μl, erythromycin (Erm) at 300 μg/μl or kanamycin (Kan) at 50 μg/ml.

**Table 3:**
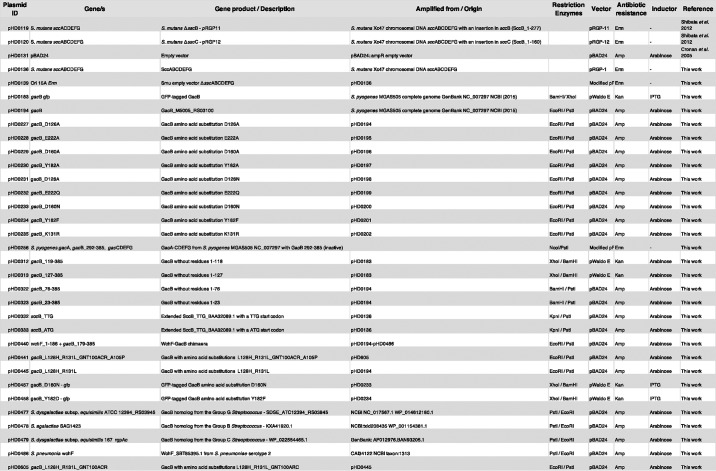
**Plasmid IDs, genes, primers, and vector details**

### Molecular genetic techniques

Table S1 shows the DNA sequence of the forward and reverse oligonucleotide primer pairs used to amplify, delete, or mutagenize the genes of interest. All primers were obtained from Integrated DNA Technologies. All PCRs were performed using a SimpliAmp Thermal Cycler from Thermo Fisher Scientific with standard procedures. Constructs were cloned using standard molecular biology procedures, including restriction enzyme digest and ligation. All constructs were validated with DNA sequencing.

### Determination of RhaPS production

Aliquots of 50 μl of *A*_600_-normalized overnight cultures grown at 37 °C were mixed with 50 μl of 6× SDS-loading buffer and resolved in 20% Tricine-SDS gels (96). Assessment of the RhaPS production was performed via immunoblotting on polyvinylidene difluoride membranes following the traditional immunoblotting technique. Primary antibody was rabbit-raised anti-*S. pyogenes* Group A carbohydrate polyclonal antibody (Abcam, ab21034). Secondary antibody was goat-raised anti-rabbit IgG horseradish peroxidase conjugate (Bio-Rad, 170-6515). Immunoreactive signals were captured using either GENESYS^TM^ 10S UV-visible spectrophotometer (Thermo Scientific) after exposure to the Clarity Western ECL (Bio-Rad).

### Extraction and radiolabeling of lipid-linked oligosaccharides

Radiolabeled LLSs of induced *E. coli* CS2775 cells bearing the selected plasmids were extracted using CHCl_3_/CH_3_OH and water-saturated butan-1-ol (1:1 v/v) solution to determine the addition of sugar residues *in vivo* after glucose D[6s^3^H] (N) (PerkinElmer Life Sciences) supplementation (1 mCi/ml). The incorporated radioactivity was measured in a Beckman LS6000SE scintillation counter. The organic phase containing the LLSs was normalized to 0.05 μCi/μl. The samples were separated via TLC on an HPTLC Silica Gel 60 plate (Merck) using a C:M:AC:A:W mobile phase (180 ml of chloroform + 140 ml of methanol + 9 ml of 1 m ammonium acetate + 9 ml of 13 m ammonia solution, 23 ml of distilled water), the plates were dried and sprayed with En3Hance liquid (PerkinElmer Life Sciences). Radioautography images were obtained using Carestream® Kodak® BioMax® XAR Film and MS intensifying screens after 5–10 days.

### Purification of recombinantly expressed membrane-associated proteins

The purification was conducted following the established protocol from Waldo *et al.* (97) with the following modifications. Overnight cultures of *E. coli* C43 (DE3) cells expressing C-terminal GFP fusion proteins were diluted 1:100, incubated for 3 h until *A*_600_ = 0.6, induced with 0.5 mm isopropyl 1-thio-β-d-galactopyranoside, and shifted to room temperature overnight, all at 200 rpm shaking. GFP expression was detected through in-gel fluorescence using a Fuji FLA-5000 laser scanner. For cloning, expression, and purification of GacB-WT, GacB-D160N-GFP, and GacB-Y182-GFP, plasmids containing GFP-His_8_-tagged recombinant proteins were constructed as described in Table 3 into the vector *p*Waldo-E (97). For protein production and purification purposes, the vectors were transformed into *E. coli* C43 (DE3) cells and expressed as described above. The cells were fractionated using an Avestin C3 high-pressure homogenizer (Biopharma) and spun down at 4000 × *g*. Further centrifugation of the supernatant at 200,000 × *g* for 2 h rendered 2–3 g of membrane containing the GacB-GFP proteins. Membranes were solubilized in Buffer 1 (500 mm NaCl, 10 mm Na_2_HPO_4_, 1.8 mm KH_2_PO_4_, 2.7 mm KCl, pH 7.4, 20 mm imidazole, 0.44 mm tris(2-carboxyethyl)phosphine) with the addition of 1% DDM (Anatrace) for 2 h at 4 °C and bound to a 1-ml nickel-Sepharose 6 Fast Flow (GE Healthcare) column, prewashed with buffer 1 plus 0.03% DDM. Elution was conducted using Buffer 1 supplemented with 250 mm imidazole and 0.03% DDM. Imidazole was removed using a HiPrep 26/10 desalting column (GE Healthcare) equilibrated with buffer (PBS, 0.03% DDM, 0.4 mm tris(2-carboxyethyl)phosphine). The GFP-His tag was removed with PreScission Protease cleavage in a 1:100 ratio overnight at 4 °C. Cleaved GacB proteins were collected after negative IMAC. Protein identity and purity was determined by tryptic peptide mass fingerprinting, matrix-assisted laser desorption/ionization TOF MS (MALDI-TOF), respectively (University of Dundee “Fingerprints” proteomics facility).

### Synthesis of acceptor 1 and 2

Acceptor 2 (P^1^-(11-phenoxyundecyl)-P^2^-(2-acetamido-2-deoxy-α-d-glucopyranosyl) diphosphate, PhO(CH_2_)_11_-PP-GlcNAc) was synthesized as sodium salt from phenoxyundecyl dihydrogen phosphate and 2-acetamido-2-deoxy-3,4,6-tri-*O*-acetyl-α-d-glucopyranosyl dihydrogen phosphate according to the procedure by Druzhinina *et al.* (98). Acceptor 1 (P^1^-tridecyl-P^2^-(2-acetamido-2-deoxy-α-d-glucopyranosyl) diphosphate, CH_3_(CH_2_)_12_-PP-GlcNAc) was synthesized from tridecyl dihydrogen phosphate (obtained similarly to phenoxyundecyl dihydrogen phosphate) by the same procedure as described for acceptor 2.

### GacB in vitro enzymatic reaction

Purified GacB-WT-GFP, GacB-D160N-GFP, GacB-Y182F-GFP, and the GacB (tagless) protein (0.15 mg/ml final concentration) were mixed in a 100-μl TBS buffer supplemented with 1 mm TDP-Rha as sugar donor and 1 mm acceptor 1 (CH_3_(CH_2_)_12_-PP-GlcNAc) or 1 mm acceptor 2 (PhO(CH_2_)_11_-PP-GlcNAc)) as acceptor substrate. The reaction was incubated for 3–24 h at 30 °C. The assay mixture was adjusted with the exchange of the nucleotide sugar donor to UDP-Rha or UDP-GlcNAc and with the addition of either 1 mm MgCl_2_, 1 mm MnCl_2_, or 1 mm EDTA to define the essentiality of metal dependence.

### MS analysis

MALDI-TOF was used to analyze the acceptors and products of the GacB *in vitro* assay. 100-μl reaction samples were purified over 100-μl Sep-Pak C18 cartridges (Waters), pre-equilibrated with 5% EtOH. The bound samples were washed with 800 μl of H_2_O and 800 μl of 15% EtOH, eluted in two fractions with (*a*) 800 μl of 30% EtOH and (*b*) 800 μl of 60% EtOH. The two elution fractions were dried in a SpeedVac and resuspended in 20 μl of 50% MeOH. 1 μl of sample was mixed with 1 μl of 2,5-dihydroxybenzoic acid matrix (15 mg/ml in 30:70 acetonitrile, 0.1% TFA), and 1 μl was added to the MALDI grid. Samples were analyzed by MALDI in an Autoflex speed mass spectrometer set up in reflection positive ion mode (Bruker, Germany).

### NMR analysis

The purified GacB *in vitro* assay products (0.5–2 mg) were dissolved in D_2_O (550 μl) and measured at 300 K. The spectra were acquired on a four-channel Avance III 800-MHz Bruker NMR spectrometer equipped with a 5-mm TCI CryoProbe^TM^ with automated matching and tuning. 1D spectra were acquired using the relaxation and acquisition times of 5 and 1.8 s, respectively. Between 32 and 512 scans were acquired using the spectral width of 11 ppm. *J* connectivities were established in a series of 1D and 2D TOCSY experiments with mixing times between 20 and 120 ms. Selective 1D TOCSY spectra (99) were acquired using 40-ms Gaussian pulses and DIPSI-2 sequence (100) (γB_1_/2π = 10 kHz) for spin lock of between 20 and 120 ms. The following parameters were used to acquire 2D TOCSY and ROESY experiments: 2048 and 768 complex points in *t*_2_ and *t*_1_, respectively, spectral widths of 11 and 8 ppm in *F*_2_ and *F*_1_, yielding *t*_2_ and *t*_1_ acquisition times of 116 and 60 ms, respectively. 16 scans were acquired for each *t*_1_ increment using a relaxation time of 1.5 s. The overall acquisition time was 6–7 h/experiment. A forward linear prediction to 4096 points was applied in *F*_1_. A zero filling to 4096 was applied in *F*_2_. A cosine square window function was used for apodization prior to Fourier transformation in both dimensions. The ROESY mixing time was applied in the form of a 250-ms rectangular pulse at γB_1_/2π = 4167 Hz. DIPSI-2 sequence (γB_1_/2π = 10 kHz) was applied for a 20, 80, and 120 ms spin lock. 2D magnitute mode heteronuclear multiple-bond correlation (HMBC) experiments were conducted using the following parameters: 2048 and 128 complex points in *t*_2_ and *t*_1_, respectively, spectral widths of 6 and 500 ppm in *F*_2_ and *F*_1_, yielding *t*_2_ and *t*_1_ acquisition times of 0.35 s and 0.6 ms, respectively. Two scans were acquired for each of 128 *t*_1_ increments using a relaxation time of 1.2 s. The overall acquisition time was 8 min. A forward linear prediction to 512 points was applied in *F*_1_; zero filling to 4096 was applied in *F*_2_. A sine square window function was used for apodization prior to Fourier transformation in both dimensions.

## Author contributions

A. Z., B. H. M., E. A., D. U., and H. C. D. conceptualization; A. Z., B. H. M., and E. A. data curation; A. Z., B. H. M., E. A., D. U., and H. C. D. formal analysis; A. Z., B. H. M., E. A., D. U., and H. C. D. validation; A. Z., B. H. M., E. A., V. I. T., V. V. V., L. L. D., D. U., and H. C. D. investigation; A. Z., B. H. M., E. A., D. U., and H. C. D. visualization; A. Z., B. H. M., E. A., V. I. T., V. V. V., L. L. D., D. U., and H. C. D. methodology; A. Z., B. H. M., E. A., V. I. T., V. V. V., D. U., and H. C. D. writing-original draft; A. Z., B. H. M., E. A., L. L. D., D. U., and H. C. D. writing-review and editing; D. U. and H. C. D. supervision; D. U. and H. C. D. funding acquisition; H. C. D. resources; H. C. D. project administration.

## Footnotes

5 Please note that the JBC is not responsible for the long-term archiving and maintenance of this site or any other third party hosted site.

## Footnotes

4 The abbreviations used are:

GAC: Group A carbohydrate
DDM: dodecyl maltoside
Rha: rhamnose
RhaPS: rhamnose polysaccharide
GroP: glycerol phosphate
TDP-Rha: TDP-β-l-rhamnose
Und: undecaprenyl
GT: glycosyltransferase
SCC: *S. mutans* serotype C carbohydrate
LLS: lipid-linked saccharide
TLC: thin-layer chromatography
1D and 2D: one- and two-dimensional, respectively
TOCSY: total correlation spectroscopy
ROESY: rotating-frame NOE
Tricine: *N*-[2-hydroxy-1,1-bis(hydroxymethyl)ethyl]glycine
GBS: GCS, and GGS, Group B, Group C, and Group G *Streptococcus* species, respectively.

## References

[1] Sims Sanyahumbi A., Colquhoun S., Wyber R., and Carapetis J. R. (2016) Global disease burden of Group A *Streptococcus*. in Streptococcus pyogenes: Basic Biology to Clinical Manifestations (Ferretti J. J., Stevens D. L., and Fischetti V. A., eds) pp. 661–704, University of Oklahoma Health Sciences Center, Oklahoma City 26866218

[2] Cunningham M. W. (2000) Pathogenesis of group A streptococcal infections. Clin. Microbiol. Rev. 13, 470–511 10.1128/CMR.13.3.470 10885988 PMC88944

[3] Ferretti J., and Köhler W. (2016) History of streptococcal research. in Streptococcus pyogenes: Basic Biology to Clinical Manifestations (Ferretti J. J., Stevens D. L., and Fischetti V. A., eds) pp. 1–26, University of Oklahoma Health Sciences Center, Oklahoma City 26866208

[4] Efstratiou A., and Lamagni T. (2016) Epidemiology of *Streptococcus pyogenes*. in Streptococcus pyogenes: Basic Biology to Clinical Manifestations (Ferretti J. J., Stevens D. L., and Fischetti V. A., eds) pp. 601–628, University of Oklahoma Health Sciences Center, Oklahoma City 26866208

[5] Sika-Paotonu D., Beaton A., Raghu A., Steer A., and Carapetis J. (2016) Acute rheumatic fever and rheumatic heart disease. in Streptococcus pyogenes: Basic Biology to Clinical Manifestations (Ferretti J. J., Stevens D. L., and Fischetti V. A., eds) pp. 771–826, University of Oklahoma Health Sciences Center, Oklahoma City

[6] Rodriguez-Iturbe B., and Haas M. (2016) Post-streptococcal glomerulonephritis. in Streptococcus pyogenes: Basic Biology to Clinical Manifestations (Ferretti J. J., Stevens D. L., and Fischetti V. A. eds) pp. 869–892, University of Oklahoma Health Sciences Center, Oklahoma City 26866208

[7] van Sorge N. M., Cole J. N., Kuipers K., Henningham A., Aziz R. K., Kasirer-Friede A., Lin L., Berends E. T. M., Davies M. R., Dougan G., Zhang F., Dahesh S., Shaw L., Gin J., Cunningham M., et al (2014) The classical Lancefield antigen of Group A *Streptococcus* is a virulence determinant with implications for vaccine design. Cell Host Microbe 15, 729–740 10.1016/j.chom.2014.05.009 24922575 PMC4078075

[8] Kristian S. A., Datta V., Weidenmaier C., Kansal R., Fedtke I., Peschel A., Gallo R. L., and Nizet V. (2005) d-Alanylation of teichoic acids promotes Group A *Streptococcus* antimicrobial peptide resistance, neutrophil survival, and epithelial cell invasion. J. Bacteriol. 187, 6719–6725 10.1128/JB.187.19.6719-6725.2005 16166534 PMC1251589

[9] Henningham A., Davies M. R., Uchiyama S., van Sorge N. M., Lund S., Chen K. T., Walker M. J., Cole J. N., and Nizet V. (2018) Virulence role of the GlcNAc side chain of the Lancefield cell wall carbohydrate antigen in non-M1-serotype Group A *Streptococcus*. mBio 9, e02294–17 29382733 10.1128/mBio.02294-17 PMC5790915

[10] Le Breton Y., Belew A. T., Freiberg J. A., Sundar G. S., Islam E., Lieberman J., Shirtliff M. E., Tettelin H., El-Sayed N. M., and McIver K. S. (2017) Genome-wide discovery of novel M1T1 group A streptococcal determinants important for fitness and virulence during soft-tissue infection. PLoS Pathog. 13, e1006584 10.1371/journal.ppat.1006584 28832676 PMC5584981

[11] Shelburne S. A. 3rd, Keith D., Horstmann N., Sumby P., Davenport M. T., Graviss E. A., Brennan R. G., and Musser J. M. (2008) A direct link between carbohydrate utilization and virulence in the major human pathogen group A *Streptococcus*. Proc. Natl. Acad. Sci. U.S.A. 105, 1698–1703 10.1073/pnas.0711767105 18230719 PMC2234207

[12] Lancefield R. C. (1933) A serological differentiation of human and other groups of hemolytic streptococci. J. Exp. Med. 57, 571–595 10.1084/jem.57.4.571 19870148 PMC2132252

[13] McCarty M. (1958) Further studies on the chemical basis for serological specificity of group a streptococcal carbohydrate. J. Exp. Med. 108, 311–323 10.1084/jem.108.3.311 13575668 PMC2136878

[14] Rush J. S., Edgar R. J., Deng P., Chen J., Zhu H., van Sorge N. M., Morris A. J., Korotkov K. V., and Korotkova N. (2017) The molecular mechanism of *N*-acetylglucosamine side-chain attachment to the Lancefield group A carbohydrate in *Streptococcus pyogenes*. J. Biol. Chem. 292, 19441–19457 10.1074/jbc.M117.815910 29021255 PMC5702681

[15] Mistou M.-Y., Sutcliffe I. C., and van Sorge N. M. (2016) Bacterial glycobiology: rhamnose-containing cell wall polysaccharides in Gram-positive bacteria. FEMS Microbiol. Rev. 40, 464–479 10.1093/femsre/fuw006 26975195 PMC4931226

[16] Coligan J. E., Kindt T. J., and Krause R. M. (1978) Structure of the streptococcal groups A, A-variant and C carbohydrates. Immunochemistry 15, 755–760 10.1016/0161-5890(78)90105-0 85600

[17] Krause R. M., and McCarty M. (1961) Studies on the chemical structure of the streptococcal cell wall. J. Exp. Med. 114, 127–140 10.1084/jem.114.1.127 13754097 PMC2137441

[18] Edgar R. J., van Hensbergen V. P., Ruda A., Turner A. G., Deng P., Le Breton Y., El-Sayed N. M., Belew A. T., McIver K. S., McEwan A. G., Morris A. J., Lambeau G., Walker M. J., Rush J. S., Korotkov K. V., et al (2019) Discovery of glycerol phosphate modification on streptococcal rhamnose polysaccharides. Nat. Chem. Biol. 15, 463–471 10.1038/s41589-019-0251-4 30936502 PMC6470023

[19] Zeleznick L. D., Boltralik J. J., Barkulis S. S., Smith C., and Heymann H. (1963) Biosynthesis of streptococcal cell walls: a rhamnose polysaccharide. Science 140, 400–401 10.1126/science.140.3565.400 14003420

[20] Heymann H., Manniello J. M., and Barkulis S. S. (1967) Structure of streptococcal cell walls. V. Phosphate esters in the walls of group A *Streptococcus pyogenes*. Biochem. Biophys. Res. Commun. 26, 486–491 10.1016/0006-291X(67)90574-8 5340505

[21] van Hensbergen V. P., Movert E., de Maat V., Lüchtenborg C., Le Breton Y., Lambeau G., Payré C., Henningham A., Nizet V., van Strijp J. A. G., Brügger B., Carlsson F., McIver K. S., and van Sorge N. M. (2018) Streptococcal Lancefield polysaccharides are critical cell wall determinants for human Group IIA secreted phospholipase A2 to exert its bactericidal effects. PLoS Pathog. 14, e1007348 10.1371/journal.ppat.1007348 30321240 PMC6201954

[22] Sewell E. W. C., and Brown E. D. (2014) Taking aim at wall teichoic acid synthesis: new biology and new leads for antibiotics. J. Antibiot. 67, 43–51 10.1038/ja.2013.100 24169797

[23] Huang D. H., Rama Krishna N., and Pritchard D. G. (1986) Characterization of the group A streptococcal polysaccharide by two-dimensional ^1^H-nuclear-magnetic-resonance spectroscopy. Carbohydr. Res. 155, 193–199 10.1016/S0008-6215(00)90145-9 3539332

[24] van der Beek S. L., Le Breton Y., Ferenbach A. T., Chapman R. N., van Aalten D. M. F., Navratilova I., Boons G.-J., McIver K. S., van Sorge N. M., and Dorfmueller H. C. (2015) GacA is essential for Group A *Streptococcus* and defines a new class of monomeric dTDP-4-dehydrorhamnose reductases (RmlD). Mol. Microbiol. 98, 946–962 10.1111/mmi.13169 26278404 PMC4832382

[25] Le Breton Y., Belew A. T., Valdes K. M., Islam E., Curry P., Tettelin H., Shirtliff M. E., El-Sayed N. M., and McIver K. S. (2015) Essential genes in the core genome of the human pathogen *Streptococcus pyogenes*. Sci. Rep. 5, 9838 10.1038/srep09838 25996237 PMC4440532

[26] Shibata Y., Yamashita Y., Ozaki K., Nakano Y., and Koga T. (2002) Expression and characterization of streptococcal rgp genes required for rhamnan synthesis in *Escherichia coli*. Infect. Immun. 70, 2891–2898 10.1128/IAI.70.6.2891-2898.2002 12010977 PMC128017

[27] Brown S., Santa Maria J. P. Jr., and Walker S. (2013) Wall teichoic acids of Gram-positive bacteria. Annu. Rev. Microbiol. 67, 313–336 10.1146/annurev-micro-092412-155620 24024634 PMC3883102

[28] Chapot-Chartier M.-P., and Kulakauskas S. (2014) Cell wall structure and function in lactic acid bacteria. Microb. Cell Fact. 13, S9 10.1186/1475-2859-13-S1-S9 25186919 PMC4155827

[29] Jankute M., Cox J. A. G., Harrison J., and Besra G. S. (2015) Assembly of the mycobacterial cell wall. Annu. Rev. Microbiol. 69, 405–423 10.1146/annurev-micro-091014-104121 26488279

[30] Magee A. D., and Yother J. (2001) Requirement for capsule in colonization by *Streptococcus pneumoniae*. Infect. Immun. 69, 3755–3761 10.1128/IAI.69.6.3755-3761.2001 11349040 PMC98386

[31] Nelson G. E., Pondo T., Toews K.-A., Farley M. M., Lindegren M. L., Lynfield R., Aragon D., Zansky S. M., Watt J. P., Cieslak P. R., Angeles K., Harrison L. H., Petit S., Beall B., and Van Beneden C. A. (2016) Epidemiology of invasive Group A streptococcal infections in the United States, 2005–2012. Clin. Infect. Dis. 63, 478–486 10.1093/cid/ciw248 27105747 PMC5776658

[32] Sadovskaya I., Vinogradov E., Courtin P., Armalyte J., Meyrand M., Giaouris E., Palussière S., Furlan S., Péchoux C., Ainsworth S., Mahony J., van Sinderen D., Kulakauskas S., Guérardel Y., and Chapot-Chartier M.-P. (2017) Another brick in the wall: a rhamnan polysaccharide trapped inside peptidoglycan of *Lactococcus lactis*. mBio 8, 10.1128/mBio.01303-17 10.1128/mBio.01303-17 28900021 PMC5596347

[33] Swoboda J. G., Campbell J., Meredith T. C., and Walker S. (2010) Wall teichoic acid function, biosynthesis, and inhibition. ChemBioChem 11, 35–45 10.1002/cbic.200900557 19899094 PMC2798926

[34] Weidenmaier C., and Peschel A. (2008) Teichoic acids and related cell-wall glycopolymers in Gram-positive physiology and host interactions. Nat. Rev. Microbiol. 6, 276–287 10.1038/nrmicro1861 18327271

[35] Moreland N. J., Waddington C. S., Williamson D. A., Sriskandan S., Smeesters P. R., Proft T., Steer A. C., Walker M. J., Baker E. N., Baker M. G., Lennon D., Dunbar R., Carapetis J., and Fraser J. D. (2014) Working towards a group A streptococcal vaccine: report of a collaborative Trans-Tasman workshop. Vaccine 32, 3713–3720 10.1016/j.vaccine.2014.05.017 24837510

[36] Courtney H. S., Ofek I., Penfound T., Nizet V., Pence M. A., Kreikemeyer B., Podbielski A., Podbielbski A., Hasty D. L., and Dale J. B. (2009) Relationship between expression of the family of M proteins and lipoteichoic acid to hydrophobicity and biofilm formation in *Streptococcus pyogenes*. PLoS One 4, e4166 10.1371/journal.pone.0004166 19132104 PMC2613554

[37] Brandenburg K., Andrä J., Müller M., Koch M. H. J., and Garidel P. (2003) Physicochemical properties of bacterial glycopolymers in relation to bioactivity. Carbohydr. Res. 338, 2477–2489 10.1016/j.carres.2003.08.008 14670710

[38] Willis L. M., and Whitfield C. (2013) Structure, biosynthesis, and function of bacterial capsular polysaccharides synthesized by ABC transporter-dependent pathways. Carbohydr. Res. 378, 35–44 10.1016/j.carres.2013.05.007 23746650

[39] Mazmanian S. K., and Kasper D. L. (2006) The love–hate relationship between bacterial polysaccharides and the host immune system. Nat. Rev. Immunol. 6, 849–858 10.1038/nri1956 17024229

[40] Ogura K., Okumura K., Shimizu Y., Kirikae T., and Miyoshi-Akiyama T. (2018) Pathogenicity induced by invasive infection of *Streptococcus dysgalactiae* subsp. *equisimilis* in a mouse model of diabetes. Front. Microbiol. 9, 2128 10.3389/fmicb.2018.02128 30298057 PMC6160533

[41] Hyams C., Camberlein E., Cohen J. M., Bax K., and Brown J. S. (2010) The *Streptococcus pneumoniae* capsule inhibits complement activity and neutrophil phagocytosis by multiple mechanisms. Infect. Immun. 78, 704–715 10.1128/IAI.00881-09 19948837 PMC2812187

[42] Chia J.-S., Lin Y.-L., Lien H.-T., and Chen J.-Y. (2004) Platelet aggregation induced by serotype polysaccharides from *Streptococcus mutans*. Infect. Immun. 72, 2605–2617 10.1128/IAI.72.5.2605-2617.2004 15102769 PMC387875

[43] De A., Liao S., Bitoun J. P., Roth R., Beatty W. L., Wu H., and Wen Z. T. (2017) Deficiency of RgpG causes major defects in cell division and biofilm formation, and deficiency of LytR-CpsA-Psr family proteins leads to accumulation of cell wall antigens in culture medium by *Streptococcus mutans*. Appl. Environ. Microbiol. 83, 00928–17 10.1128/AEM.00928-17 28687645 PMC5561293

[44] Tsuda H., Yamashita Y., Toyoshima K., Yamaguchi N., Oho T., Nakano Y., Nagata K., and Koga T. (2000) Role of serotype-specific polysaccharide in the resistance of *Streptococcus mutans* to phagocytosis by human polymorphonuclear leukocytes. Infect. Immun. 68, 644–650 10.1128/IAI.68.2.644-650.2000 10639428 PMC97187

[45] Caliot É., Dramsi S., Chapot-Chartier M.-P., Courtin P., Kulakauskas S., Péchoux C., Trieu-Cuot P., and Mistou M.-Y. (2012) Role of the Group B antigen of *Streptococcus agalactiae*: a peptidoglycan-anchored polysaccharide involved in cell wall biogenesis. PLoS Pathog. 8, e1002756 10.1371/journal.ppat.1002756 22719253 PMC3375309

[46] St. Michael F., Yang Q., Cairns C., Vinogradov E., Fleming P., Hayes A. C., Aubry A., and Cox A. D. (2018) Investigating the candidacy of the serotype specific rhamnan polysaccharide based glycoconjugates to prevent disease caused by the dental pathogen *Streptococcus mutans*. Glycoconj. J. 35, 53–64 10.1007/s10719-017-9798-z 28971282

[47] Kovacs C. J., Faustoferri R. C., and Quivey R. G. (2017) RgpF is required for maintenance of stress tolerance and virulence in *Streptococcus mutans*. J. Bacteriol. 199, e00497–17 10.1128/JB.00497-17 28924033 PMC5686612

[48] Nagata E., Okayama H., Ito H.-O., Yamashita Y., Inoue M., and Oho T. (2006) Serotype-specific polysaccharide of *Streptococcus mutans* contributes to infectivity in endocarditis. Oral Microbiol. Immunol. 21, 420–423 10.1111/j.1399-302X.2006.00317.x 17064403

[49] Rubens C. E., Wessels M. R., Heggen L. M., and Kasper D. L. (1987) Transposon mutagenesis of type III group B *Streptococcus*: correlation of capsule expression with virulence. Proc. Natl. Acad. Sci. U.S.A. 84, 7208–7212 10.1073/pnas.84.20.7208 2823254 PMC299259

[50] Bruyere T., Wachsmann D., Klein J. P., Schöller M., and Frank R. M. (1987) Local response in rat to liposome-associated *Streptococcus mutans* polysaccharide-protein conjugate. Vaccine 5, 39–42 10.1016/0264-410X(87)90007-7 3577355

[51] Cartee R. T., Forsee W. T., Bender M. H., Ambrose K. D., and Yother J. (2005) CpsE from type 2 *Streptococcus pneumoniae* catalyzes the reversible addition of glucose-1-phosphate to a polyprenyl phosphate acceptor, initiating type 2 capsule repeat unit formation. J. Bacteriol. 187, 7425–7433 10.1128/JB.187.21.7425-7433.2005 16237026 PMC1272991

[52] Charbonneau A. R. L., Forman O. P., Cain A. K., Newland G., Robinson C., Boursnell M., Parkhill J., Leigh J. A., Maskell D. J., and Waller A. S. (2017) Defining the ABC of gene essentiality in streptococci. BMC Genomics 18, 426 10.1186/s12864-017-3794-3 28569133 PMC5452409

[53] Endo A., and Rothfield L. (1969) Studies of a phospholipid-requiring bacterial enzyme. I. Purification and properties of uridine diphosphate galactose: lipopolysaccharide α-3-galactosyl transferase. Biochemistry 8, 3500–3507 10.1021/bi00837a003 4898284

[54] Wollin R., Creeger E. S., Rothfield L. I., Stocker B. A., and Lindberg A. A. (1983) *Salmonella typhimurium* mutants defective in UDP-D-galactose:lipopolysaccharide α1,6-d-galactosyltransferase: structural, immunochemical, and enzymologic studies of rfaB mutants. J. Biol. Chem. 258, 3769–3774 6403519

[55] Marchler-Bauer A., Bo Y., Han L., He J., Lanczycki C. J., Lu S., Chitsaz F., Derbyshire M. K., Geer R. C., Gonzales N. R., Gwadz M., Hurwitz D. I., Lu F., Marchler G. H., Song J. S., et al (2017) CDD/SPARCLE: functional classification of proteins via subfamily domain architectures. Nucleic Acids Res. 45, D200–D203 10.1093/nar/gkw1129 27899674 PMC5210587

[56] Martinez-Fleites C., Proctor M., Roberts S., Bolam D. N., Gilbert H. J., and Davies G. J. (2006) Insights into the synthesis of lipopolysaccharide and antibiotics through the structures of two retaining glycosyltransferases from family GT4. Chem. Biol. 13, 1143–1152 10.1016/j.chembiol.2006.09.005 17113996

[57] Vetting M. W., Frantom P. A., and Blanchard J. S. (2008) Structural and enzymatic analysis of MshA from *Corynebacterium glutamicum*: substrate-assisted catalysis. J. Biol. Chem. 283, 15834–15844 10.1074/jbc.M801017200 18390549 PMC2414306

[58] Lairson L. L., Henrissat B., Davies G. J., and Withers S. G. (2008) Glycosyltransferases: structures, functions, and mechanisms. Annu. Rev. Biochem. 77, 521–555 10.1146/annurev.biochem.76.061005.092322 18518825

[59] Persson K., Ly H. D., Dieckelmann M., Wakarchuk W. W., Withers S. G., and Strynadka N. C. (2001) Crystal structure of the retaining galactosyltransferase LgtC from *Neisseria meningitidis* in complex with donor and acceptor sugar analogs. Nat. Struct. Biol. 8, 166–175 10.1038/84168 11175908

[60] Wang S., Tanaka H., Hindsgaul O., Lam J. S., and Brockhausen I. (2013) A convenient synthesis of GDP-d-rhamnose: the donor substrate for d-rhamnosyltransferase WbpZ from *Pseudomonas aeruginosa* PAO1. Bioorg. Med. Chem. Lett. 23, 3491–3495 10.1016/j.bmcl.2013.04.051 23664878

[61] Franco O. L., and Rigden D. J. (2003) Fold recognition analysis of glycosyltransferase families: further members of structural superfamilies. Glycobiology 13, 707–712 10.1093/glycob/cwg098 12881407

[62] Ozaki K., Shibata Y., Yamashita Y., Nakano Y., Tsuda H., and Koga T. (2002) A novel mechanism for glucose side-chain formation in rhamnose-glucose polysaccharide synthesis. FEBS Lett. 532, 159–163 10.1016/S0014-5793(02)03661-X 12459482

[63] Lehrer J., Vigeant K. A., Tatar L. D., and Valvano M. A. (2007) Functional characterization and membrane topology of *Escherichia coli* WecA, a sugar-phosphate transferase initiating the biosynthesis of enterobacterial common antigen and O-antigen lipopolysaccharide. J. Bacteriol. 189, 2618–2628 10.1128/JB.01905-06 17237164 PMC1855806

[64] Albesa-Jové D., Giganti D., Jackson M., Alzari P. M., and Guerin M. E. (2014) Structure–function relationships of membrane-associated GT-B glycosyltransferases. Glycobiology 24, 108–124 10.1093/glycob/cwt101 24253765 PMC3907083

[65] Klena J. D., and Schnaitman C. A. (1994) Genes for TDP-rhamnose synthesis affect the pattern of lipopolysaccharide heterogeneity in *Escherichia coli* K-12. J. Bacteriol. 176, 4003–4010 10.1128/jb.176.13.4003-4010.1994 7517388 PMC205598

[66] Pradel E., Parker C. T., and Schnaitman C. A. (1992) Structures of the rfaB, rfaI, rfaJ, and rfaS genes of *Escherichia coli* K-12 and their roles in assembly of the lipopolysaccharide core. J. Bacteriol. 174, 4736–4745 10.1128/jb.174.14.4736-4745.1992 1624461 PMC206270

[67] Villegas A., and Kropinski A. M. (2008) An analysis of initiation codon utilization in the domain Bacteria: concerns about the quality of bacterial genome annotation. Microbiol. 154, 2559–2661 10.1099/mic.0.2008/021360-0 18757789

[68] Wittgens A., Santiago-Schuebel B., Henkel M., Tiso T., Blank L. M., Hausmann R., Hofmann D., Wilhelm S., Jaeger K.-E., and Rosenau F. (2018) Heterologous production of long-chain rhamnolipids from *Burkholderia glumae* in *Pseudomonas putida*—a step forward to tailor-made rhamnolipids. Appl. Microbiol. Biotechnol. 102, 1229–1239 10.1007/s00253-017-8702-x 29264775

[69] Jurtshuk P. (1996) Bacterial metabolism. in Medical Microbiology, 4th Ed. (Baron S., ed) University of Texas Medical Branch at Galveston, Galveston, TX 21413278

[70] Chang A., Singh S., Phillips G. N. Jr., and Thorson J. S. (2011) Glycosyltransferase structural biology and its role in the design of catalysts for glycosylation. Curr. Opin. Biotechnol. 22, 800–808 10.1016/j.copbio.2011.04.013 21592771 PMC3163058

[71] Breton C., Snajdrová L., Jeanneau C., Koca J., and Imberty A. (2006) Structures and mechanisms of glycosyltransferases. Glycobiology 16, 29R–37R 10.1093/glycob/cwj016 16037492

[72] Parsonage D., Newton G. L., Holder R. C., Wallace B. D., Paige C., Hamilton C. J., Dos Santos P. C., Redinbo M. R., Reid S. D., and Claiborne A. (2010) Characterization of the *N*-acetyl-α-d-glucosaminyl l-malate synthase and deacetylase functions for bacillithiol biosynthesis in *Bacillus anthracis*. Biochemistry 49, 8398–8414 20799687 10.1021/bi100698n PMC2943542

[73] Lombard V., Golaconda Ramulu H., Drula E., Coutinho P. M., and Henrissat B. (2014) The carbohydrate-active enzymes database (CAZy) in 2013. Nucleic Acids Res. 42, D490–D495 10.1093/nar/gkt1178 24270786 PMC3965031

[74] Breton C., Fournel-Gigleux S., and Palcic M. M. (2012) Recent structures, evolution and mechanisms of glycosyltransferases. Curr. Opin. Struct. Biol. 22, 540–549 10.1016/j.sbi.2012.06.007 22819665

[75] De Bruyn A., and Anteunis M. (1976) ^1^H-N.m.r. study of L-rhamnose, methyl α-l-rhamnopyranoside, and 4-*o*-β-d-galactopranosyl-l-rhamnose in deuterium oxide. Carbohydr. Res. 47, 158–163 10.1016/S0008-6215(00)83559-4 1268870

[76] Schäffer C., Wugeditsch T., Kählig H., Scheberl A., Zayni S., and Messner P. (2002) The surface layer (S-layer) glycoprotein of *Geobacillus stearothermophilus* NRS 2004/3a: analysis of its glycosylation. J. Biol. Chem. 277, 6230–6239 10.1074/jbc.M108873200 11741945

[77] Hong Y., and Reeves P. R. (2014) Diversity of *o*-antigen repeat unit structures can account for the substantial sequence variation of wzx translocases. J. Bacteriol. 196, 1713–1722 10.1128/JB.01323-13 24532778 PMC3993327

[78] Ristl R., Steiner K., Zarschler K., Zayni S., Messner P., and Schäffer C. (2011) The S-layer glycome—adding to the sugar coat of bacteria. Int. J. Microbiol. 2011, 127870 10.1155/2011/127870 20871840 PMC2943079

[79] Wang S., Hao Y., Lam J. S., Vlahakis J. Z., Szarek W. A., Vinnikova A., Veselovsky V. V., and Brockhausen I. (2015) Biosynthesis of the common polysaccharide antigen of *Pseudomonas aeruginosa* PAO1: characterization and role of GDP-d-rhamnose: GlcNAc/GalNAc-diphosphate-lipid α1,3-d-rhamnosyltransferase WbpZ. J. Bacteriol. 197, 2012–2019 10.1128/JB.02590-14 25845842 PMC4438205

[80] Rubirés X., Saigi F., Piqué N., Climent N., Merino S., Albertí S., Tomás J. M., and Regué M. (1997) A gene (wbbL) from *Serratia marcescens* N28b (O4) complements the rfb-50 mutation of *Escherichia coli* K-12 derivatives. J. Bacteriol. 179, 7581–7586 10.1128/jb.179.23.7581-7586.1997 9393727 PMC179713

[81] Dhaked D. K., Bala Divya M., and Guruprasad L. (2019) A structural and functional perspective on the enzymes of *Mycobacterium tuberculosis* involved in the l-rhamnose biosynthesis pathway. Prog. Biophys. Mol. Biol. 145, 52–64 10.1016/j.pbiomolbio.2018.12.004 30550737

[82] Michon F., Brisson J. R., Dell A., Kasper D. L., and Jennings H. J. (1988) Multiantennary group-specific polysaccharide of group B *Streptococcus*. Biochemistry 27, 5341–5351 10.1021/bi00414a059 3048399

[83] Pritchard D. G., and Furner R. L. (1985) Structure of the group-specific polysaccharide of group E *Streptococcus*. Carbohydr. Res. 144, 289–296 10.1016/S0008-6215(00)90676-1 4092208

[84] James D. B. A., and Yother J. (2012) Genetic and biochemical characterizations of enzymes involved in *Streptococcus pneumoniae* serotype 2 capsule synthesis demonstrate that Cps2T (WchF) catalyzes the committed step by addition of β1–4 rhamnose, the second sugar residue in the repeat unit. J. Bacteriol. 194, 6479–6489 10.1128/JB.01135-12 23002227 PMC3497468

[85] Islam S. T., and Lam J. S. (2013) Wzx flippase-mediated membrane translocation of sugar polymer precursors in bacteria. Environ. Microbiol. 15, 1001–1015 10.1111/j.1462-2920.2012.02890.x 23016929

[86] Islam S. T., and Lam J. S. (2014) Synthesis of bacterial polysaccharides via the Wzx/Wzy-dependent pathway. Can. J. Microbiol. 60, 697–716 10.1139/cjm-2014-0595 25358682

[87] Bentley S. D., Aanensen D. M., Mavroidi A., Saunders D., Rabbinowitsch E., Collins M., Donohoe K., Harris D., Murphy L., Quail M. A., Samuel G., Skovsted I. C., Kaltoft M. S., Barrell B., Reeves P. R., et al (2006) Genetic analysis of the capsular biosynthetic locus from all 90 pneumococcal serotypes. PLoS Genet. 2, e31 10.1371/journal.pgen.0020031 16532061 PMC1391919

[88] Nothaft H., and Szymanski C. M. (2010) Protein glycosylation in bacteria: sweeter than ever. Nat. Rev. Microbiol. 8, 765–778 10.1038/nrmicro2383 20948550

[89] Rini J., Esko J., and Varki A. (2009) Glycosyltransferases and glycan-processing enzymes. in Essentials of Glycobiology, 2nd Ed. (Varki A., Cummings R. D., Esko J. D., Freeze H. H., Stanley P., Bertozzi C. R., Hart G. W., and Etzler M. E., eds), Cold Spring Harbor Laboratory Press, Cold Spring Harbor, NY 20301247

[90] Guerin M. E., Schaeffer F., Chaffotte A., Gest P., Giganti D., Korduláková J., van der Woerd M., Jackson M., and Alzari P. M. (2009) Substrate-induced conformational changes in the essential peripheral membrane-associated mannosyltransferase PimA from mycobacteria: implications for catalysis. J. Biol. Chem. 284, 21613–21625 10.1074/jbc.M109.003947 19520856 PMC2755885

[91] Sutcliffe I. C., Black G. W., and Harrington D. J. (2008) Bioinformatic insights into the biosynthesis of the Group B carbohydrate in *Streptococcus agalactiae*. Microbiol. Read. Engl. 154, 1354–1363 10.1099/mic.0.2007/014522-0 18451044

[92] Clarke A. J., Hurtado-Guerrero R., Pathak S., Schüttelkopf A. W., Borodkin V., Shepherd S. M., Ibrahim A. F. M., and van Aalten D. M. F. (2008) Structural insights into mechanism and specificity of *O*-GlcNAc transferase. EMBO J. 27, 2780–2788 10.1038/emboj.2008.186 18818698 PMC2556091

[93] Ding J., Pan X., Du L., Yao Q., Xue J., Yao H., Wang D.-C., Li S., and Shao F. (2019) Structural and functional insights into host death domains inactivation by the bacterial arginine GlcNAcyltransferase effector. Mol. Cell 74, 922–935.e6 10.1016/j.molcel.2019.03.028 30979585

[94] Sievers F., Wilm A., Dineen D., Gibson T. J., Karplus K., Li W., Lopez R., McWilliam H., Remmert M., Söding J., Thompson J. D., and Higgins D. G. (2011) Fast, scalable generation of high-quality protein multiple sequence alignments using Clustal Omega. Mol. Syst. Biol. 7, 539–539 10.1038/msb.2011.75 21988835 PMC3261699

[95] Letunic I., and Bork P. (2016) Interactive tree of life (iTOL) v3: an online tool for the display and annotation of phylogenetic and other trees. Nucleic Acids Res. 44, W242–W245 10.1093/nar/gkw290 27095192 PMC4987883

[96] Schägger H. (2006) Tricine-SDS-PAGE. Nat. Protoc. 1, 16–22 10.1038/nprot.2006.4 17406207

[97] Waldo G. S., Standish B. M., Berendzen J., and Terwilliger T. C. (1999) Rapid protein-folding assay using green fluorescent protein. Nat. Biotechnol. 17, 691–695 10.1038/10904 10404163

[98] Druzhinina T. N., Danilov L. L., Torgov V. I., Utkina N. S., Balagurova N. M., Veselovsky V. V., and Chizhov A. O. (2010) 11-Phenoxyundecyl phosphate as a 2-acetamido-2-deoxy-α-d-glucopyranosyl phosphate acceptor in O-antigen repeating unit assembly of *Salmonella arizonae* O:59. Carbohydr. Res. 345, 2636–2640 10.1016/j.carres.2010.09.021 20974465

[99] Robinson P. T., Pham T. N., and Uhrín D. (2004) In phase selective excitation of overlapping multiplets by gradient-enhanced chemical shift selective filters. J. Magn. Reson. 170, 97–103 10.1016/j.jmr.2004.06.004 15324762

[100] Rucker F. J., and Osorio D. (2008) The effects of longitudinal chromatic aberration and a shift in the peak of the middle-wavelength sensitive cone fundamental on cone contrast. Vision Res. 48, 1929–1939 10.1016/j.visres.2008.06.021 18639571 PMC2773461

[101] Artimo P., Jonnalagedda M., Arnold K., Baratin D., Csardi G., de Castro E., Duvaud S., Flegel V., Fortier A., Gasteiger E., Grosdidier A., Hernandez C., Ioannidis V., Kuznetsov D., Liechti R., et al (2012) ExPASy: SIB bioinformatics resource portal. Nucleic Acids Res. 40, W597–W603 10.1093/nar/gks400 22661580 PMC3394269

[102] Viklund H., and Elofsson A. (2008) OCTOPUS: improving topology prediction by two-track ANN-based preference scores and an extended topological grammar. Bioinformatics 24, 1662–1668 10.1093/bioinformatics/btn221 18474507

[103] Kelley L. A., Mezulis S., Yates C. M., Wass M. N., and Sternberg M. J. (2015) The Phyre2 web portal for protein modeling, prediction and analysis. Nat. Protoc. 10, 845–858 10.1038/nprot.2015.053 25950237 PMC5298202

